# Supplementary material for: How does social desirability bias influence survey-based estimates of the use of antenatal care in rural Nepal? A validation study
Source: BMJ Open. 2023 Jul 26;13(7):e071511. doi: 10.1136/bmjopen-2022-071511 (PMC10373690; doi:10.1136/bmjopen-2022-071511)
Supplement: Supplementary data [file bmjopen-2022-071511supp001.pdf]

Supplementary Materials

Supplementary Figure 1. Study flow chart

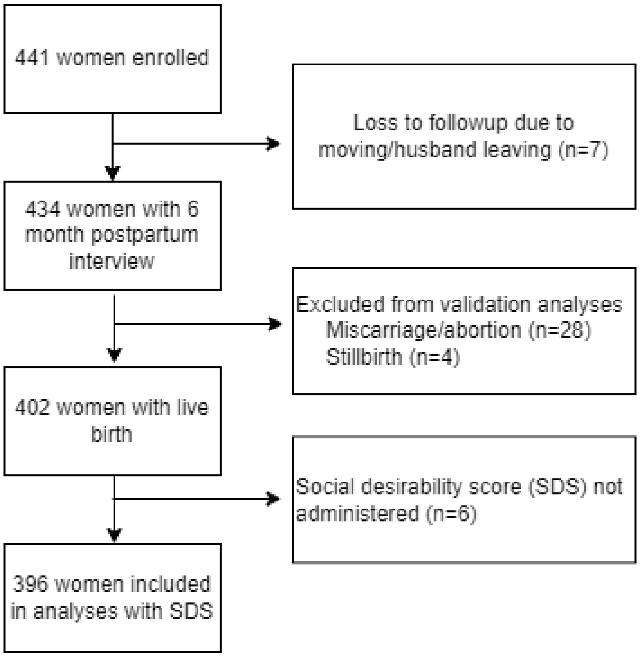

Supplementary Tables

Supplementary Table 1. Questions included in the survey and their origins[1]

Question	Responses	Origin/notes
21. How many times did you receive antenatal care during this pregnancy?	Number of times:<br>01-15 times (go to 21.a.)<br>99= Don't know (go to 22)	2016 Demographic and Health Survey, DHS-8
23. During this pregnancy, were you given or did you buy any iron/folic acid tablets?<br><br>Show photo of tablets	0= No (go to 24)<br>1= Yes (go to 23.a.)<br>9= Don't Know (go to 24)	2016 Demographic and Health Survey, DHS-8
23.a. During the whole pregnancy, for how many days did you take the tablets?<br>If answer is not numeric, probe for approximate number of days	Days 000-270<br>999=Don't know	2016 Demographic and Health Survey, DHS-8
27. During this pregnancy, at any antenatal visit, did you receive any information about nutrition or diet?	0= No (go to 28)<br>1= Yes (go to 27.a.)<br>9= Don't Know (go to 28)	Original question
27.b. What information or messages did you receive during your pregnancy about nutrition or diet?<br><br>First allow respondent to provide any answers and check all that apply<br><br>Then probe to identify each response and record all mentioned<br>[ask each answer individually]	Were you told to…<br><br>□ Eat more (quantity)<br>□ Eat a variety of foods rich with iron, vitamin A or C<br>□ Take iron tablets (IFA's)<br>□ Take calcium tablets<br>□ How to manage nausea/vomiting<br>□ Other (specify) ______<br><br>0= No<br>1= Yes, without prompting<br>2= Yes, with prompting<br>9= Don't know	Original question

[1]These questions were administered to respondents approximately 6 months after delivery, asking them to recall services received during the most recent pregnancy. The responses column lists the possible response categories for each question and the origin column describes the source of the question.

Supplementary Table 2. List of questions included from the short form Marlowe Crown social desirability index, inclusion in the score

Code	Question	% Yes	Negative key[1]
SM1	Do you sometimes find it hard to go on with your work if you are not encouraged?	39.6	No
SM2	Do you sometimes feel resentful when you don't get your way?	10.0	No
SM3	Do you occasionally give up doing something because you don't think you have the ability	31.1	No
SM4	Are there any times when you felt like rebelling against people in authority even though you knew they were right?	59.9	No
SM5	Are you always a good listener no matter who you are talking to?	21.9	Yes
SM6	Are there any occasions when you took advantage of someone?	89.1	No
SM7	When you make a mistake are you always willing to admit it?	64.7	Yes
SM8	Are you always courteous, even to people who are disagreeable/not pleasant?	19.5	Yes
SM9	Have you ever been irked when people expressed ideas very different from your own?	14.4	No
SM10	Are there any times when you were quite jealous of the good fortune of others?	85.1	No
SM11	Do you sometimes get irritated/annoyed by people who ask you to do something for them?	27.6	No
SM12	Have you ever deliberately said something that hurt someone's feelings	89.5	No

Supplementary Table 3. Correlations between questions used for assessment of the social desirability

		SM1	SM2	SM3	SM4	SM5	SM6	SM7	SM8	SM9	SM10	SM11	SM12
SM1	Do you sometimes find it hard to go on with your work if you are not encouraged?	1.00											
SM2	Do you sometimes feel resentful when you don't get your way?	0.31 ***	1.00										
SM3	Do you occasionally give up doing something because you don't think you have the ability	0.07	0.08	1.00									
SM4	Are there any times when you felt like rebelling against people in authority even though you knew they were right?	-0.03	0.10 *	-0.08	1.00								
SM5	Are you always a good listener no matter who you are talking to?	0.15 **	0.16 **	0.03	0.22 ***	1.00							
SM6	Are there any occasions when you took advantage of someone?	.02	.01	-0.01	0.11 *	-0.03	1.00						
SM7	When you make a mistake are you always willing to admit it?	.08	0.04	-0.12 *	0.36 ***	0.23 ***	-0.04	1.00					
SM8	Are you always courteous, even to people who are disagreeable/not pleasant?	.21 ***	0.21 ***	0.03	0.03	0.38 ***	-0.03	0.21 ***	1.00				
SM9	Have you ever been irked when people expressed ideas very different from your own?	.20 ***	0.34 ***	-0.03	0.15 **	0.16 **	0.07	0.13 *	0.32 ***	1.00			
SM10	Are there any times when you were quite jealous of the good fortune of others?	0.16 **	0.07	0.04	0.15 *	0.07	0.06	0.15 **	0.05	0.11 *	1.00		
SM11	Do you sometimes get irritated/annoyed by people who ask you to do something for them?	.29 ***	0.34 ***	0.01	0.15 **	0.28 ***	-0.02	0.21 ***	0.32 ***	0.29 ***	0.15 **	1.00	
SM12	Have you ever deliberately said something that hurt someone's feelings	-0.11 *	- 0.02	0.005	0.02	0.07	0.17 **	- 0.04	-0.02	-0.07	0.06	0.03	1.00

***p<0.0001, **p<0.01 *p<0.05

Supplementary Table 4. Characteristics of women and households by presence of others during the interview

	No other adults present (n=195)	Any other adult present (n=207)	Husband present (n=28)	Mother in law present (n=98)	Friend present (n=50)	Overall (n=402)
Women's age, years (mean, sd)	22.7 (4.3)	22.3 (4.1)	20.6 (2.9)	21.8 (3.7)	23.4 (4.8)	22.5 (4.2)
# Observed ANC visits	5.0 (2.6)	4.3 (2.3)	4. 1 (2.1)	4.4 (2.5)	4.4 (2.3)	4.7 (2.5)
Socioeconomic status quartile %						
1 (Bottom)	34.9	38.7	32.1	36.7	40.0	36.8
2	18.0	17.4	21.4	17.4	16.0	17.7
3	31.3	31.4	32.1	29.6	38.0	31.3
4 (Top)	15.9	12.6	14.3	16.3	6.0	14.2
Any prior live births (%)	70.3	67.2	60.7	59.2	74.0	68.7
Any formal education (%)	42.1	38.6	42.9	44.9	24.0	40.3
Trimester at enrollment						
1-3 months	46.2	38.7	28.6	40.8	34.0	42.3
4-6 months	51.8	59.9	71.4	57.1	66.0	56.0
7-9 months	2.1	1.5	0	2.0	0	1.7
Got help during interview*	0	52.0	50.0	48.1	32.7	19.9

Supplementary Table 5. Receipt of help during the interview and accuracy of recall of services

	Received help during interview from another adult ARR (95% CI)	Received help from MIL during the interview ARR (95% CI)
Deworming	0.97 (0.78, 1.20)	0.94 (0.78, 1.13)
Receipt of Ca or Ca info	0.99 (0.82, 1.20)	1.01 (0.90, 1.13)
Counseling on		
Nutrition	0.97 (0.85, 1.11)	No convergence
Eating more	0.93 (0.77, 1.12)	No convergence
Diverse diet	No convergence	No convergence
Managing nausea	1.02 (0.70, 1.49)	1.18 (0.91, 1.53)
Not drinking *paan* alcohol	0.94 (0.64, 1.38)	1.24 (1.00, 1.55)
Not smoking/ using	0.88 (0.60, 1.29)	1.14 (0.92, 1.41)
Accuracy of IFA estimate		
Within 30 tablets	1.08 (0.69, 1.69)	1.16 (0.81, 1.64)
Within 60 tablets	1.04 (0.80, 1.36)	1.07 (0.85, 1.34)
Within 90 tablets	1.05 (0.89, 1.24)	1.09 (0.96, 1.25)
Estimate number of ANC visits		
Exactly	0.47 (0.16, 1.38)	0.76 (0.39, 1.49)
Within 1 visit	0.86 (0.57, 1.29)	0.81 (0.58, 1.13)
Within 2 visits	0.98 (0.78, 1.23)	1.02 (0.86, 1.21)
