# Supplementary material for: How does social desirability bias influence survey-based estimates of the use of antenatal care in rural Nepal? A validation study
Source: BMJ Open. 2023 Jul 26;13(7):e071511. doi: 10.1136/bmjopen-2022-071511 (PMC10373690; doi:10.1136/bmjopen-2022-071511)
Supplement: Author's manuscript [file bmjopen-2022-071511.draft_revisions.pdf]

BMJ Open

BMJ Open is committed to open peer review. As part of this commitment we make the peer review history of every article we publish publicly available.

When an article is published we post the peer reviewers' comments and the authors' responses online. We also post the versions of the paper that were used during peer review. These are the versions that the peer review comments apply to.

The versions of the paper that follow are the versions that were submitted during the peer review process. They are not the versions of record or the final published versions. They should not be cited or distributed as the published version of this manuscript.

BMJ Open is an open access journal and the full, final, typeset and author-corrected version of record of the manuscript is available on our site with no access controls, subscription charges or pay-per-view fees (http://bmjopen.bmj.com).

If you have any questions on BMJ Open's open peer review process please email info.bmjopen@bmj.com

BMJ Open

Social desirability bias when estimating use of antenatal care services in Nepal

Journal:	*BMJ Open*
Manuscript ID	bmjopen-2022-071511
Article Type:	Original research
Date Submitted by the Author:	17-Jan-2023
Complete List of Authors:	Thorne-Lyman, Andrew; Johns Hopkins University Bloomberg School of Public Health, International Health<br>Plama, Tsering; Johns Hopkins University Bloomberg School of Public Health, International Health; Nepal Nutrition Intervention Project-Sarlahi<br>Heidkamp, Rebecca; Johns Hopkins University Bloomberg School of Public Health, International Health<br>Munos, Melinda; Johns Hopkins University, Bloomberg School of Public Health, Department of International Health<br>Manandhar, Porcia; Johns Hopkins University Bloomberg School of Public Health, International Health<br>Khatry, Subarna; Nepal Nutr Intervent Project Sarlahi; Johns Hopkins University Bloomberg School of Public Health, International Health<br>Bryce, Emily; Johns Hopkins University Bloomberg School of Public Health, International Health<br>LeClerq, Steven C.; Johns Hopkins Bloomberg School of Public Health, Department of International Health; Nepal Nutrition Intervention Project<br>Katz, Joanne; Johns Hopkins University Bloomberg School of Public Health, International Health
Keywords:	Nutrition < TROPICAL MEDICINE, PRIMARY CARE, PUBLIC HEALTH, OBSTETRICS

SCHOLARONE™
Manuscripts

BMJ

I, the Submitting Author has the right to grant and does grant on behalf of all authors of the Work (as defined in the below author licence), an exclusive licence and/or a non-exclusive licence for contributions from authors who are: i) UK Crown employees; ii) where BMJ has agreed a CC-BY licence shall apply, and/or iii) in accordance with the terms applicable for US Federal Government officers or employees acting as part of their official duties; on a worldwide, perpetual, irrevocable, royalty-free basis to BMJ Publishing Group Ltd ("BMJ") its licensees and where the relevant Journal is co-owned by BMJ to the co-owners of the Journal, to publish the Work in this journal and any other BMJ products and to exploit all rights, as set out in our licence.

The Submitting Author accepts and understands that any supply made under these terms is made by BMJ to the Submitting Author unless you are acting as an employee on behalf of your employer or a postgraduate student of an affiliated institution which is paying any applicable article publishing charge ("APC") for Open Access articles. Where the Submitting Author wishes to make the Work available on an Open Access basis (and intends to pay the relevant APC), the terms of reuse of such Open Access shall be governed by a Creative Commons licence – details of these licences and which Creative Commons licence will apply to this Work are set out in our licence referred to above.

Other than as permitted in any relevant BMJ Author's Self Archiving Policies, I confirm this Work has not been accepted for publication elsewhere, is not being considered for publication elsewhere and does not duplicate material already published. I confirm all authors consent to publication of this Work and authorise the granting of this licence.

For peer review only

Title: Social desirability bias when estimating use of antenatal care services in Nepal

Authors: Andrew L-Thorne-Lyman[a], Tsering Pema Lama[a,b], Rebecca A. Heidkamp[a], Melinda K. Munos[a], Porcia Manandhar[a], Subarna K. Khatry[a,b], Emily Bryce[a] Steven C. LeClerq[a,b], Joanne Katz[a]

[a]Department of International Health, Johns Hopkins Bloomberg School of Public Health, Baltimore, MD, 21205, USA

[b]Nepal Nutrition Intervention Project-Sarlahi, Kathmandu, Nepal

Corresponding author: Andrew L. Thorne-Lyman, Program in Human Nutrition, Department of International Health, 615 N. Wolfe Street E2545, Johns Hopkins Bloomberg School of Public Health, Baltimore, Maryland, USA. **Email:** athorne1@jhu.edu, **Phone**: +1 856 214-5755, **Fax**:+1 410-955-0196

Sources of support: Bill & Melinda Gates Foundation INV-007332 and OPP1172551

Competing interests: None

Keywords: Nepal, survey, nutrition, social desirability bias, antenatal care, recall, validity

For peer review only - http://bmjopen.bmj.com/site/about/guidelines.xhtml

Abstract

Introduction

Large scale surveys are used to assess the coverage of antenatal care (ANC) and nutrition interventions. Social desirability bias could affect the validity of questions about the receipt of care and coverage estimates, though this has not been well studied.

Methods

A longitudinal cohort design was used comparing direct observation of receipt of ANC and 6 specific nutrition components of ANC including different counselling and iron-folic acid against reports of receipt using a post-partum survey (n=401). The presence of socially desirable responses was assessed using a short form Marlowe-Crowne social desirability index and the presence of other interview observers as a separate exposure. Log-binomial multivariable regression was used to predict accuracy and overestimation of recalled receipt of ANC services against observed receipt adjusting for age, education, first-pregnancy, and socio-economic status.

Results

The social desirability score did not significantly predict accuracy or overestimation of most types of ANC care except counselling on nausea: higher social desirability scores were associated with more accurate recall [adjusted RR, aRR 1.08 (95% CI 1.03, 1.12)] and less overestimation, [aRR 0.85 (0.80, 0.91)]. Mothers' interviews with their mothers-in-law or husbands present were more likely to overestimate the number of ANC visits they received by more than 3 visits [aRR 2.07 (1.11, 3.84)] and [aRR 4.19 (2.17, 8.10)] respectively. Those interviewed with friends present tended to overestimate the receipt of counselling on nausea, avoiding alcohol, and not smoking.

Conclusion

For peer review only

For peer review only - http://bmjopen.bmj.com/site/about/guidelines.xhtml

Findings suggest that the presence of observers can lead to overestimation of the receipt of ANC care and support efforts to interview in private settings. Findings that the social desirability score did not predict the accuracy of most types of ANC care might reflect a reality that such questions may not be sensitive from a social-norms perspective.

Key messages

What is already known on this topic:

Social desirability bias is one of the most common forms of bias in surveys and can reduce the validity of survey findings.

Previous studies from Nepal have found that receipt of iron-folic acid supplementation is often overestimated by women compared with the gold standard of observed receipts and that validity of recall of nutrition-related interventions and counselling during ANC varies by indicator.

What this study adds:

This is the first study to directly examine whether social desirability bias, measured using a social desirability scale as well as the presence of interviewers during a survey, explains the overestimation and lack of accuracy of maternal reports of ANC receipt.

We found that presence of interview observers does lead to overestimation of the receipt of certain types of care but that the social desirability scale did not predict the accuracy of estimation of most forms of care.

How this study may affect research, practice or policy:

Large-scale surveys measuring coverage should avoid interviewing participants in the presence of interview observers.

For peer review only - http://bmjopen.bmj.com/site/about/guidelines.xhtml

Introduction

The World Health Organization (WHO) recommends a number of nutrition interventions in pregnancy, including iron-folic acid supplements (IFA), counselling on healthy eating and physical activity, and in certain contexts calcium, micronutrient supplements and balanced energy and protein supplements.[1] Large-scale household surveys, including those implemented by the Demographic Health Survey program, are used across low and middle-income countries to measure coverage of antenatal interventions in order to monitor progress towards targets and evaluate policies and programs.[2]

Many survey-based coverage indicators come from self-reported data and a growing number of studies have explored the validity of responses to questions about the receipt of nutrition interventions delivered through antenatal care.[2][3] A study carried out by our research team in Nepal found that respondents tend to overestimate the receipt of IFA in pregnancy, and that overestimation was not explained by either maternal or household characteristics.[4] Women's reports of the receipt of various counselling interventions, weight measurement, and deworming were generally recalled more accurately than questions about other services such as counselling to not drink alcohol or tobacco.[5]

Social desirability response bias (SDB) is a common type of bias that reflects the tendency of respondents to reply in a manner that may be viewed positively by social peers or that is consistent with social norms and expectations. Social desirability bias (SDB) is generally thought to be greater for questions that are perceived as sensitive or subject to judgment such as those related to sexual practices or family planning.[6,7] The perceived relationships between respondents and interviewers or observers can also influence socially desirable responses. For example, a study administered in 14 African countries found that respondents give systematically different answers to interviewers depending on whether or not they were from their same ethnic group.[8]

For peer review only - http://bmjopen.bmj.com/site/about/guidelines.xhtml

Survey-based scales to measure social desirability were developed in the field of social psychology in the 1960's beginning with the 33-question Marlowe-Crowne scale[9]. Shorter versions of these scales have been shown to be reliable across different cultural contexts, though more work is needed to refine and test them.[7,10].[11] These scales include a variety of positively keyed questions that are "culturally acceptable but improbable" or "desirable but rare" and negatively keyed items that are "culturally unacceptable but probable" or "undesirable but common".[10] The scales are used to detect and quantify the extent of socially desirable responses in surveys. Where it has been detected, a number of approaches exist correct for it or mitigate its effects including a posteriori statistical adjustment for SDS,[12,13] excluding high SDS scorers[13] or rephrasing questions using forgiving wording[14].

Social desirability bias has been frequently described as an area of potential concern in nutrition-related studies collecting data about diet, physical activity, and self-reported weight, but few studies have directly quantified the strength of association between social desirability scores and these domains.[15] Evaluations of the Alive & Thrive infant and young child feeding program in Vietnam and Bangladesh explored whether adjusting for short-form social desirability scales affected estimates of program impact on diet, but found that results were unchanged.[16,17] To our knowledge, no previous studies have directly explored whether SDB is a potential explanation for inaccurate survey responses about the receipt of ANC and related nutrition interventions. The aims of this study were therefore to:

1. Adapt and test a modified short-form version of the Marlow-Crowne social desirability scale in rural Nepal.
2. Explore whether social desirability or the presence of other adults during interviews helps to explain previous findings related to overestimation of the receipt of services.
3. Explore whether social desirability or the presence of other adults during interviews is associated with inaccurate recall of ANC and associated services.

For peer review only - http://bmjopen.bmj.com/site/about/guidelines.xhtml

Methods

Study area and population

This study took place in Sarlahi District, an area in the southern plains (*Tarai)* of Nepal located in Province 2, the province with the lowest rates of ANC coverage in Nepal. According to the 2016 Demographic and Health Survey, only 36% of women in Province 2 received all four recommended ANC visits, far below the national average of 59%.[18]. These low rates appear paradoxical given that 70% of the population in Sarlahi lived within 30 minutes of a government health facility. This paradox may be explained in part by low rates of maternal education and gender norms that limit women's ability to travel outside of the household.[19] The population of Sarlahi is linguistically and culturally heterogeneous, including both Nepali and Maithili-speaking groups.[20]

Antenatal care and nutrition services delivered through ANC

At the time of our study, Nepal was implementing the World Health Organization's focused ANC (FANC) model, with recommended visits occurring in the 4th, 6th, 8^{th,} and 9th months of pregnancy. The government incentivizes ANC participation by providing 800 rupees (~7 USD) to women who complete all four visits.[21] Nutrition interventions delivered through ANC in Nepal include the provision of iron-folic acid supplements, checking of weight, and counselling on various topics related to nutrition and breastfeeding.

Study design

This study was part of a larger effort to validate coverage indicators for various nutrition services through ANC, described in detail elsewhere.[4,5] In brief, study staff were based at five government health posts and trained to directly observe and record the receipt of ANC services by enrolled women. The observers' 28-item checklist included details about the provision of iron-folic acid pills (e.g. how

For peer review only - http://bmjopen.bmj.com/site/about/guidelines.xhtml

many pills women received, whether they were instructed to purchase iron folic acid tablets or syrup), measurement of weight, and types of counselling on nutrition and breastfeeding. These direct observations served as a "gold standard" to validate maternal recall of ANC services received at study clinics. Maternal recall was assessed by interviewing enrolled women using a structured questionnaire around six months after delivery. [2,4,5] The interview questions were drawn from the official translated Nepali DHS 2016 questionnaire (DHS Round 7), a draft of the DHS Round 8 questionnaire, and novel questions to assess different components of counselling and weight gain assessment (**Supplementary Table 1)**. The six-month visits were conducted at the women's homes or their parental homes. Women were asked about socioeconomic status at enrollment to the validation study and during the visit at six months after delivery. As described in detail elsewhere [4], a target sample size of 300 women was established for the study based on an assumption of 50% coverage for receipt of IFA and lower coverage for counselling. After accounting for loss-to-follow-up, the possibility of women going elsewhere for ANC, and of non-live birth outcomes, a target study enrollment of 450 women was established.

Measurement of social desirability

Questions from a short-form Marlowe-Crowne social desirability index (**Supplementary Table 2)** were translated and back-translated by Nepali/English speakers familiar with the study context, pre-tested and slightly adapted to the local context. During pre-testing one of the questions was not well understood and dropped; 12 questions were included in the six-month visit questionnaire.[11] Study staff also recorded whether any other adult had been present at any point during the interview (with specific codes for husbands, mothers-in law, friends of the respondent or other adults), as well as whether those present had helped the respondent to answer any questions.

For peer review only - http://bmjopen.bmj.com/site/about/guidelines.xhtml

Correlations between different items of the social desirability index were explored after reverse coding negatively keyed questions, and most were found to be positively associated with one another (**Supplementary Table 3**). Tests of internal consistency revealed the full 12-item scale to have a Cronbach's alpha of 0.58 and only minor improvements (to an alpha of 0.65) were possible by removing several items so the full 12-item scale was used in analyses. On visual inspection, scores on the social desirability scale (SDS) were only slightly skewed, with a mean of 5.5 and a standard deviation of 2.1.

Data collection

Enrollment criteria for the parent trial included currently pregnant, married women aged 15 years or older, who a) resided in the study area, b) visited one of the five health posts for their first ANC visit and c) had the intention to return to these study sites for subsequent ANC visits. All interview questions were translated and back-translated by Nepali/English speakers familiar with the study context. These questions were pre-tested and minor changes were made to help facilitate comprehension in the local context. Both Maithili and Nepali-speaking interviewers conducted the interviews following training exercises designed to standardize the approach to translation.

The study started in December 2018 and reached full enrollment by November 2019. Direct observations of ANC visits were nearly completed by March 2020 when a shut-down for the Covid-19 pandemic interrupted all non- emergency health services in Nepal. Data collection for the post-partum six-month visit was started in September 2019 but Covid-19 related shutdowns caused delays. The mean number of days that passed from delivery to the post-partum data collection visit was 252 with a standard deviation of 75 days. Precautions taken to prevent the spread of Covid-19 included masking by interviewers and respondents, handwashing with soap provided to interviewees, holding interviews outdoors when weather permitted, and maintaining a distance of more than six feet during data collection.

For peer review only - http://bmjopen.bmj.com/site/about/guidelines.xhtml

Data analysis

We examined whether SDS or presence of other adults during interviews was associated with the accuracy or overestimation of recalled receipt of specific ANC services using log-binomial bivariate and multivariable regressions or log-Poisson models in the case of model non-convergence. Multivariable analyses adjusted for age (continuous), education (any vs. none), first pregnancy (yes/no), and quartile of socioeconomic status as possible confounders. For interpretation purposes, high SDS corresponds to women that would be more likely to be exhibit socially desirable responses. SDS was treated as both a continuous score and as a dichotomous variable in analyses, with the upper quartile defined as those with a score of 7 or greater compared against the lower three quartiles, recognizing that socially desirable response bias may not be a linear construct. For receipt of counselling interventions, calcium, and deworming, accurate recall was defined as alignment (yes/no) of the follow-up interview responses with observed receipt or non-receipt of those interventions.[4,5] Overestimation for these interventions was defined as respondents that reported receipt but were not observed receiving it, and for each intervention women were categorized as over- estimators or non-over-estimators. For ANC visits and IFA, several different thresholds were used to define accuracy and the degree of overestimation. For reasons of statistical power, we restricted our analysis to questions about interventions with directly observed coverage below 90%. A complete case analysis was used for the treatment of missing data and loss to follow-up was minimal (**Supplementary Figure 1**).

IRB approval

This research conformed with the principles embodied in the Declaration of Helsinki. The institutional review boards at Johns Hopkins Bloomberg School of Public Health and the Nepal Health Research Council both approved this study including permission to collect and resume data collection during the Covid-19 epidemic. Signed consent was obtained from all participants. Patients and the public were not

For peer review only - http://bmjopen.bmj.com/site/about/guidelines.xhtml

involved in the design, conduct, reporting or dissemination plans of this research. Dissemination and discussion of study design and results is made to a national committee and a local district committee.

Results

Of the 441 women enrolled in the study, 396 were included in our analysis after accounting for exclusion criteria described in **Supplementary Figure 1**. The mean age, number of observed ANC visits, and birth history were similar among women in the upper quartile of SDS compared those in the lower three quartiles (**Table 1)**. Women with lower SDS tended to have lower education and enrolled in the study later in pregnancy than those with higher scores. Twice as many women with lower SDS had another adult present during the interview than those with higher scores; mothers-in-law and other adults were the most common observers. Those with lower SDS were only slightly more likely to have an observer help them respond to interview questions.

Table 1. Characteristics of the study population

	Bottom 3 quartiles SDS (n=284)	Upper quartile SDS (n=112)	Overall (n=396)
Age, years, mean (sd)	22.7 (4.3)	21.9 (4.0)	22.5 (4.2)
# Observed ANC visits, mean (sd)	4.6 (2.4)	4.8 (2.6)	4.7 (2.5)
Socioeconomic status quartile (%)			
1 (Bottom)	36.6	39.3	37.4
2	17.6	17.0	17.4
3	31.7	29.5	31.1
4 (Top)	14.1	14.3	14.1
Any prior live births (%)	71.7	71.8	71.7
Any formal education (%)	32.2	22.3	28.3
Trimester at enrollment (%)			
1-3 months	39.4	50.0	42.4
4-6 months	59.2	47.3	55.8
7-9 months	1.4	2.7	1.8
Interview observed by another adult (%)	60.9	31.1	52.1
Husband	7.4	5.9	7.0
Mother-in-law	28.3	14.3	24.4
Friend	15.9	5.0	12.9
Other adult	39.5	16.0	32.9

For peer review only

For peer review only - http://bmjopen.bmj.com/site/about/guidelines.xhtml

Observer helped respondent answer questions (%)	29.0	23.5	27.9

About half of women overestimated the number of IFA tablets they received by at least 30 tablets and over a third overestimated the number of ANC visits by at least one visit (**Table 2**). About a third of women reported receiving counselling for nausea, alcohol or smoking that had not been observed at clinics. Null relations were apparent between SDS and overestimation of receipt of most services, although many coefficients were positive when treating SDS as a dichotomous predictor. Additionally, the dichotomous SDS had a stronger relation with extreme overestimation of reported receipt of both ANC and IFA compared to more modest overestimation. SDS were generally not associated with accuracy of recalled services (**Table 3**). The only exception was for counselling on the management of nausea: women with higher SDS tended to have more accurate recall.

Table 2. High social desirability score as a predictor of overestimation of nutrition/ANC services receipt in pregnancy

	**N**	**Proportion Overestimating %**	**SDS[2] as a predictor of overestimation**	
			**Dichotomous SDS adjusted[1,3] RR (95% CI)**	**Continuous SDS adjusted RR[1,3] (95% CI)**
Deworming	395	15.2	1.12 (0.67, 1.87)	1.01 (0.90, 1.13)
Receipt or prescribe Ca	390	7.6	0.76 (0.33, 1.75)	0.90 (0.75, 1.07)
Counselling on				
Managing nausea	391	38.3	0.49 (0.34, 0.72)	0.85 (0.80, 0.91)
Not drinking alcohol	395	37.7	1.26 (0.97, 1.63)	1.04 (0.98, 1.10)
Not smoking/ using *paan*	395	31.2	1.23 (0.91, 1.66)	1.02 (0.95, 1.09)
Overestimate IFA receipt				
By >30 tablets	404	51.7	1.07 (0.87, 1.30)	1.02 (0.97, 1.06)
By >60 tablets	404	34.0	1.16 (0.87, 1.54)	1.03 (0.97, 1.10)
By >90 tablets	404	17.5	1.28 (0.83, 1.98)	1.02 (0.93, 1.13)
Overestimate # of ANC visits				
By >1 visit	402	34.8	0.99 (0.74, 1.34)	1.00 (0.94, 1.07)
By >2 visits	402	18.7	1.17 (0.75, 1.82)	1.02 (0.92, 1.13)
By >3 visits	402	9.2	1.37 (0.71, 2.62)	1.02 (0.87, 1.18)

[1]Adjusted risk ratio > 1 indicates greater risk of overestimating receipt of services

[2]SDS=Social desirability score, treated as upper quartile vs lower three quartiles

For peer review only

For peer review only - http://bmjopen.bmj.com/site/about/guidelines.xhtml

[3]Adjusted for woman's age (continuous), any education, first pregnancy, and socio-economic status quartile

Table 3. High social desirability score as a predictor of accurate recall of nutrition/ANC services receipt in pregnancy

	N	Proportion accurate %	SDS[2] as a predictor of accurate recall	
			Dichotomous SDS adjusted[1,3] RR (95% CI)	Continuous SDS adjusted RR[1,3] (95% CI)
Deworming	395	77.5	0.97 (0.86, 1.09)	0.99 (0.96, 1.01)
Receipt or prescribe Ca	390	85.6	0.98 (0.89, 1.09)	0.99 (0.97, 1.01)
Counselling on				
Managing nausea	391	50.9	1.30 (1.07, 1.60)	1.08 (1.03, 1.12)
Not drinking alcohol	395	55.7	0.89 (0.72, 1.10)	0.97 (0.93, 1.02)
Not smoking/ using *paan*	395	59.5	0.91 (0.74, 1.11)	0.98 (0.94, 1.02)
Overestimate IFA receipt				
By >30 tablets	396	39.9	0.87 (0.66, 1.16)	0.98 (0.93, 1.04)
By >60 tablets	396	61.6	1.01 (0.85, 1.20)	1.02 (0.98, 1.05)
By >90 tablets	396	79.3	0.91 (0.80, 1.03)	1.00 (0.97, 1.02)
Overestimate # of ANC visits				
By >1 visit	396	21.0	0.94 (0.60, 1.45)	1.01 (0.92, 1.11)
By >2 visits	396	55.1	0.91 (0.74, 1.12)	1.02 (0.97, 1.06)
By >3 visits	396	75.5	0.96 (0.84, 1.10)	1.01 (0.98, 1.04)

[1]Adjusted risk ratio > 1 indicates more accurate recall

[2]SDS=Social desirability score, treated as upper quartile vs lower three quartiles

[3]Adjusted for woman's age (continuous), any education, first pregnancy, and socio-economic status quartile

The presence of any other adult was significantly associated with overestimation of receipt of counselling on managing nausea as well as overestimation of the number of ANC visits. Mothers-in-law (MILs) were present during about a quarter of all interviews. Their presence was associated with significant overestimation of the receipt of deworming tablets [ARR 1.90 (95% C.I. 1.18, 3.05)] and more than doubled the risk of overestimating the number of ANC visits by 3 or more visits [ARR 2.07 (95% 1.11, 3.84)] **(Table 4)**. Interestingly, the presence of a MIL during the interview appeared to attenuate overestimation of IFA receipt by >90 tablets, though this estimate included the null (ARR: 0.56 (95% CI 0.31, 1.01). The presence of a husband was less common (7%) and was significantly associated with the

For peer review only

For peer review only - http://bmjopen.bmj.com/site/about/guidelines.xhtml

overestimation of ANC visits by one, two and three visits. The presence of a friend was significantly associated with overestimation of the receipt of multiple types of ANC services including information about calcium, managing nausea, not drinking alcohol and not smoking or using tobacco.

Table 4. Presence of others during the interview as a predictor of overestimation of recalled services receipt

	N	Mother in law present ARR[2] (95% CI)	Husband present ARR[2] (95% CI)	Friend present ARR[2] (95% CI)	Any adult present ARR[2] (95% CI)
Deworming	401	1.90 (1.18, 3.05)	0.50 (0.13, 1.94)	0.85 (0.41, 1.76)	1.05 (0.66, 1.67)
Receipt or prescribe Ca	396	1.02 (0.45, 2.32)	1.63 (0.52, 5.06)	2.69 (1.30, 5.55)	1.91 (0.92, 3.97)
Counselling on					
Managing nausea	397	1.17 (0.89, 1.54)	1.09 (0.68, 1.76)	1.53 (1.15, 2.04)	1.70 (1.30, 2.22)
Not drinking alcohol	401	0.80 (0.58, 1.11)	1.07 (0.66, 1.74)	1.41 (1.05, 1.90)	1.09 (0.85, 1.39)
Not smoking/ using paan	401	0.71 (0.48, 1.06)	1.22 (0.73, 2.06)	1.55 (1.12, 2.15)	0.98 (0.74, 1.31)
Overestimate IFA receipt					
By >30 tablets	402	0.94 (0.76, 1.17)	0.88 (0.60, 1.29)	0.93 (0.69, 1.25)	0.96 (0.80, 1.15)
By >60 tablets	402	0.86 (0.62, 1.19)	1.13 (0.72, 1.77)	0.89 (0.58, 1.37)	0.95 (0.73, 1.24)
By >90 tablets	402	0.56 (0.31, 1.01)	0.90 (0.40, 2.02)	0.96 (0.51, 1.81)	0.73 (0.49, 1.11)
Overestimate # of ANC visits					
By >1 visit	400	1.28 (0.97, 1.70)	1.48 (1.03, 2.13)	1.18 (0.82, 1.70)	1.33 (1.01, 1.73)
By >2 visits	400	1.32 (0.85, 2.05)	1.97 (1.13, 3.44)	1.24 (0.70, 2.20)	1.57 (1.03, 2.40)
By >3 visits	400	2.07 (1.11, 3.84)	4.19 (2.17, 8.10)	0.43 (0.10, 1.72)	2.26 (1.15, 4.46)

[1]Adjusted risk ratio > 1 indicates greater risk of overestimation of reported services receipt

[3]Adjusted for woman's age (continuous), any education, first pregnancy, and socio-economic status quartile

Women interviewed in the presence of MILs were less accurate in estimating the number of ANC visits but more accurate in recalling the receipt of counselling related to avoiding alcohol consumption in pregnancy. Women tended to be less accurate when estimating the number of ANC visits or receipt of IFA in front of their husbands. (**Table 5**).

For peer review only - http://bmjopen.bmj.com/site/about/guidelines.xhtml

Table 5 Presence of others during the interview as a predictor of accuracy of recalled services receipt

	N	Mother in law present RR (95% CI)	Husband present RR (95% CI)	Friend present RR (95% CI)	Any adult present RR (95% CI)
Deworming	401	0.91 (0.79, 1.04)	1.13 (0.99, 1.29)	0.84 (0.57, 1.25)	1.03 (0.93, 1.14)
Receipt or prescribe Ca	396	1.03 (0.94, 1.12)	0.97 (0.81, 1.16)	0.87 (0.74, 1.04)	0.99 (0.92, 1.08)
Counselling on					
Managing nausea	397	0.94 (0.74, 1.19)	0.78 (0.49, 1.25)	0.71 (0.50, 1.02)	0.72 (0.59, 0.87)
Not drinking alcohol	401	1.20 (1.00, 1.45)	1.07 (0.79, 1.45)	0.78 (0.55, 1.10)	1.01 (0.85, 1.20)
Not smoking/ using paan	401	1.14 (0.95, 1.35)	0.91 (0.66, 1.27)	0.76 (0.55, 1.05)	1.04 (0.89, 1.22)
Accuracy of IFA estimate					
Within 30 tablets	402	0.96 (0.73, 1.28)	1.06 (0.68, 1.68)	1.17 (0.84, 1.63)	1.01 (0.80, 1.29)
Within 60 tablets	402	0.91 (0.75, 1.11)	0.76 (0.50, 1.14)	0.96 (0.76, 1.20)	0.94 (0.81, 1.09)
Within 90 tablets	402	1.00 (0.89, 1.12)	0.69 (0.50, 0.96)	0.96 (0.82, 1.13)	0.99 (0.90, 1.10)
Estimate # of ANC visits					
Exactly	400	0.67 (0.41, 1.10)	0.47 (0.16, 1.40)	1.25 (0.75, 2.08)	0.94 (0.65, 1.36)
Within 1 visit	400	0.86 (0.69, 1.08)	0.87 (0.59, 1.27)	1.07 (0.83, 1.37)	1.04 (0.87, 1.24)
Within 2 visits	400	0.85 (0.74, 0.99)	0.76 (0.56, 1.04)	1.07 (0.92, 1.24)	0.93 (0.82, 1.04)

[1]Adjusted risk ratio > 1 indicates greater accuracy of reported services receipt

[3]Adjusted for woman's age (continuous), any education, first pregnancy, and socio-economic status quartile

We also examined how the receipt of help from observers to answer questions during the interview may have affected the accuracy of recall of services **(Supplementary Table 5)**. Assistance from MIL was associated with more accurate recall of counselling to not drink alcohol [ARR 1.24 (95% C.I. 1.00, 1.55) but no other services. In contrast, assistance from husbands did not significantly affect the accuracy of recall of any services.

Discussion

Previous studies exploring the validity of self-reported questions about the receipt of ANC care have raised social desirability bias as a possible type of error that might affect coverage estimates.[22,23] To our knowledge, however, this is the first study in the peer-reviewed literature to directly explore whether social desirability influences the validity of women's recalled receipt of ANC services. We found that SDS did not significantly predict the accuracy of women's recall of most types of

For peer review only - http://bmjopen.bmj.com/site/about/guidelines.xhtml

ANC services, except for counselling on the management of nausea. However, the presence of another adult observer during the interview did influence the accuracy of recall. Women that were interviewed with their MIL or husbands present tended to overestimate the number of ANC visits they had received and those interviewed with friends present tended to overestimate the receipt of different types of counselling.

The effects of social desirability bias are complex[24] and socially desirable responses may be driven by either unconscious distortions related to self-deception or impression management related to social norms.[13,25] The potential for social desirability bias to affect questions about nutrition and receipt of ANC services in low- and middle-income countries has only been indirectly explored in the literature. For example, a study conducted in China found that recalled receipt of Hepatitis B blood tests (HBsAb) was much higher than HIV tests despite similar observed coverage and suggested that the difference might be explained by socially desirable responses.[22] Many of the questions that we tested in our study related to whether or not women had received specific nutrition interventions as part of ANC visits. A likely explanation for null associations with the SDS is that such questions do not reflect practices that might be perceived negatively (or positively) from the standpoint of social norms.[24] It is unclear why higher scores on the SDS were positively associated with more accurate recall of counselling on management of nausea. One possible explanation is that the counselling was focused only women that complained of nausea and not all women.[5] Thus, it is possible that the socially desirable response to this question was to accurately respond to whether or not counselling had been received, and that these responses were particularly easy to recall because they were related to recollections of nausea.

The finding that the presence of MILs and/or husbands during the interview was associated with a significantly greater overestimation of the number of ANC visits is consistent with the hypothesis that social norms in this context may support more frequent visits for ANC. Cognitive testing[26] conducted in this same population revealed that women were confused about whether visits to providers outside

For peer review only - http://bmjopen.bmj.com/site/about/guidelines.xhtml

of health posts including for sonograms should be counted or not, and it is possible that women might be more likely to count such visits when in the presence of other household members.

IFA coverage is an important indicator for global monitoring of nutrition interventions; it is included in the Global Nutrition Monitoring Framework and an indicator for the Countdown to 2030. [27,28] There are global concerns about accuracy of recall of the number of tablets received or consumed during a specific pregnancy, particularly in the DHS or other surveys where respondents may be 2-5 years postpartum. Previous work in Nepal showed that at six months post-partum women's self-reported receipt of IFA tended to overestimate the number of supplements that they actually received and that this overestimation was not associated with maternal characteristics including age, education, parity, or SES.[4]

Our study is the first that we are aware of to test a social desirability scale in the context of rural Nepal, though SD scales have been used in urban India[24] and rural Bangladesh.[17] We pretested scale items and made some modifications. The reliability coefficient of our tested scale of 0.58 neared the threshold of 0.60 used to define acceptability, and marginally surpassed that threshold when two items were removed. These values were slightly less than reliability coefficients documented in a study using a lengthier version of the Marlowe-Crowne SDS in four African countries, which varied from 0.63 (Kenya) to 0.80 (Ethiopia).[7] Many of the scale items in our study were positively correlated, however, it is uncertain how well the questions were understood by respondents or the extent to which the scale reflects social desirability in the context of rural Nepal. Additional work to develop and test questions that draw more strongly on local situations and norms would likely further strengthen the ability of the scale to capture social desirability in rural contexts, similar to what has been developed in the Philippines,[29](2) and could enhance the utility of such scales for use in surveys.

For peer review only

For peer review only - http://bmjopen.bmj.com/site/about/guidelines.xhtml

314 A strength of our study is that we used direct observation to assess the receipt of services in clinics. However, the tool used to assess social desirability has not been validated in a rural context, and was only slightly adapted for comprehension by respondents in our area. It is not clear how well the scale was able to capture socially desirable response tendencies in this context. Our study used a recall period of approximately 6 months, which may have facilitated more accurate recall of receipt than typically is assessed in surveys such as the DHS, which currently administers ANC modules to women up to 2-years postpartum. The implications of different recall periods for social desirability bias are unclear. Additionally, the enumerators noted the presence of others at any point in the interview, and not specifically during the questions about receipt of services which would be likely to attenuate estimates of the relationship between the presence of others and validity.

324 These findings reinforce the guidance often given during survey training to conduct surveys without others present, though doing so in practice is often challenging in rural village settings where norms around privacy differ. As we found that the social desirability scale was not well correlated with overestimation or lack of accurate recall, there was no need to correct for social desirability bias. Given that the generalizability of our study to other contexts including other parts of Nepal is uncertain, we recommend further studies be conducted in other contexts to develop and validate social desirability scales and use them to better understand the effects of social desirability bias on estimates of services use.

332 **Acknowledgements**

333 The Nepal Nutrition Intervention Project Sarlahi operates with the permission of and under the auspices of the Social Welfare Council in Nepal and in close conjunction with Nepal Netra Jyoti Singh. This work was funded by the Bill & Melinda Gates Foundation INV007332 and OPP1172551.

For peer review only

Contributorship statement

Conceived study design: MKM, JK, ATL, RAH; Implemented the study: TPL, SL, SKK, EB, PM; Analyzed the data: ATL; Wrote the paper: ATL; Read, provided edits and approved final manuscript: all authors. The authors thank the study team at the Nepal Nutrition Intervention Project-Sarlahi, who conducted household data collection. The Nepal Nutrition Intervention Project Sarlahi operates with the permission of and under the auspices of the Social Welfare Council in Nepal and in close conjunction with Nepal Netra Jyoti Singh.

Competing interests

The authors declare no competing interests in relation to this work

Funding

This work was funded by the Bill & Melinda Gates Foundation INV007332, OPP1174256 and OPP1172551.

Data sharing statement

Date will be made available on reasonable request

Ethics approval statement

This study was approved by the institutional review board at Johns Hopkins Bloomberg School of Public Health and by the Nepal Health Research Council. Participants gave informed consent to participate in the study.

References

1 World Health Organization. *WHO recommendations on antenatal care for a positive pregnancy experience*. World Health Organization 2016.

For peer review only

For peer review only - http://bmjopen.bmj.com/site/about/guidelines.xhtml

2 Munos MK, Blanc AK, Carter ED, *et al.* Validation studies for population-based intervention coverage indicators: design, analysis, and interpretation. *J Glob Health* 2018;**8**.

3 Hill Z, Okyere E, Wickenden M, *et al.* What can we learn about postnatal care in Ghana if we ask the right questions? A qualitative study. *Glob Health Action* 2015;**8**:28515.

4 Bryce E, Katz J, Munos MK, *et al.* Validation of Maternal Recall of Iron Folic Supplementation During Antenatal Care in Rural Southern Nepal. *Curr Dev Nutr* 2021;**5**:722–722.

5 Bryce E, Katz J, Heidkamp R, *et al.* Validation of maternal report of nutrition-related interventions and counselling during antenatal care in southern Nepal. *Matern Child Nutr* 2021;:e13303.

6 Stuart GS, Grimes DA. Social desirability bias in family planning studies: a neglected problem. *Contraception* 2009;**80**:108–12.

7 Vu A, Tran N, Pham K, *et al.* Reliability of the Marlowe-Crowne social desirability scale in Ethiopia, Kenya, Mozambique, and Uganda. *BMC Med Res Methodol* 2011;**11**:1–7.

8 Adida CL, Ferree KE, Posner DN, *et al.* Who's asking? Interviewer coethnicity effects in African survey data. *Comp Polit Stud* 2016;**49**:1630–60.

9 Crowne DP, Marlowe D. A new scale of social desirability independent of psychopathology. *J Consult Psychol* 1960;**24**:349.

10 Cagasan Jr L. The development and validation of a Filipino social desirability scale. *Philipp J Psychol* 2016;**49**:19–42.

11 Reynolds WM. Development of reliable and valid short forms of the Marlowe-Crowne Social Desirability Scale. *J Clin Psychol* 1982;**38**:119–25.

12 Paulhus DL. Measurement and control of response bias. 1991.

13 Larson RB. Controlling social desirability bias. *Int J Mark Res* 2019;**61**:534–47.

14 Näher A-F, Krumpal I. Asking sensitive questions: the impact of forgiving wording and question context on social desirability bias. *Qual Quant* 2012;**46**:1601–16.

15 Westerterp KR, Goris AH. Validity of the assessment of dietary intake: problems of misreporting. *Curr Opin Clin Nutr Metab Care* 2002;**5**:489–93.

16 Nguyen PH, Kim SS, Sanghvi T, *et al.* Integrating nutrition interventions into an existing maternal, neonatal, and child health program increased maternal dietary diversity, micronutrient intake, and exclusive breastfeeding practices in Bangladesh: results of a cluster-randomized program evaluation. *J Nutr* 2017;**147**:2326–37.

17 Menon P, Nguyen PH, Saha KK, *et al.* Impacts on breastfeeding practices of at-scale strategies that combine intensive interpersonal counselling, mass media, and community mobilization: results of cluster-randomized program evaluations in Bangladesh and Viet Nam. *PLoS Med* 2016;**13**:e1002159.

For peer review only

For peer review only - http://bmjopen.bmj.com/site/about/guidelines.xhtml

18 Ministry of Health, Nepal; New ERA; and ICF. Nepal Demographic and Health Survey 2016. Kathmandu, Nepal: : Ministry of Health, Nepal 2017.

19 Adhikari M, Chalise B, Bista B, *et al.* Sociodemographic correlates of antenatal care visits in Nepal: results from Nepal Demographic and Health Survey 2016. *BMC Pregnancy Childbirth* 2020;**20**:1–10.

20 Mullany LC, Katz J, Li YM, *et al.* Breast-feeding patterns, time to initiation, and mortality risk among newborns in southern Nepal. *J Nutr* 2008;**138**:599–603.

21 World Health Organization. WHO antenatal care randomized trial: manual for the implementation of the new model. World Health Organization 2002.

22 Liu L, Li M, Yang L, *et al.* Measuring coverage in MNCH: a validation study linking population survey derived coverage to maternal, newborn, and child health care records in rural China. *PLoS One* 2013;**8**:e60762.

23 Benova L, Tunçalp Ö, Moran AC, *et al.* Not just a number: examining coverage and content of antenatal care in low-income and middle-income countries. *BMJ Glob Health* 2018;**3**:e000779.

24 Fisher RJ, Katz JE. Social-desirability bias and the validity of self-reported values. *Psychol Mark* 2000;**17**:105–20.

25 Crowne DP, Crowne DP, Marlowe D. *The approval motive: Studies in evaluative dependence*. New York: Wiley 1964.

26 Thorne-Lyman AL, Lama TP, Heidkamp RA, *et al.* Cognitive testing of questions about antenatal care and nutrition interventions in southern Nepal. *Soc Sci Med* In Press.

27 World Health Organization. Developing and validating an iron and folic acid supplementation indicator for tracking progress towards global nutrition monitoring framework targets: final report June 2018. 2018.

28 Boerma T, Requejo J, Victora CG, *et al.* Countdown to 2030: tracking progress towards universal coverage for reproductive, maternal, newborn, and child health. *The Lancet* 2018;**391**:1538–48.

29 Ranjan A, George TS. Evaluating social desirability bias in assessment. *Indian J Health Wellbeing* 2014;**5**:948.

For peer review only

For peer review only - http://bmjopen.bmj.com/site/about/guidelines.xhtml

Supplementary Materials

Supplementary Figure 1. Study flow chart

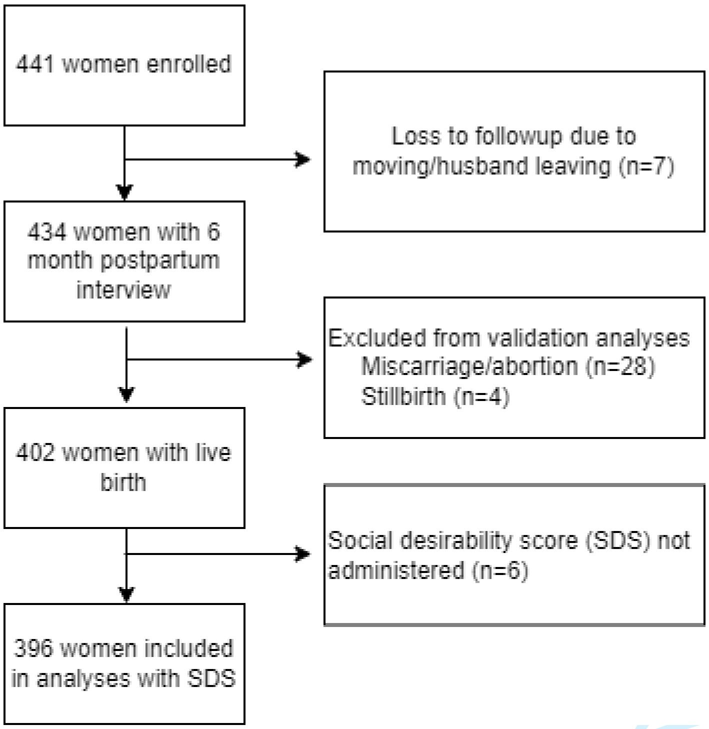

Supplementary Tables

Supplementary Table 1. Questions included in the survey and their origins

**Question**	**Responses**	**Origin/notes**
21. How many times did you receive antenatal care during this pregnancy?	Number of times:<br>01-15 times (go to 21.a.)<br>99= Don't know (go to 22)	2016 Demographic and Health Survey, DHS-8
23. During this pregnancy, were you given or did you buy any iron/folic acid tablets?<br><br>Show photo of tablets	0= No (go to 24)<br>1= Yes (go to 23.a.)<br>9= Don't Know (go to 24)	2016 Demographic and Health Survey, DHS-8
23.a. During the whole pregnancy, for how many days did you take the tablets?<br>If answer is not numeric, probe for approximate number of days	Days 000-270<br>999=Don't know	2016 Demographic and Health Survey, DHS-8
27. During this pregnancy, at any antenatal visit, did you receive any information about nutrition or diet?	0= No (go to 28)<br>1= Yes (go to 27.a.)<br>9= Don't Know (go to 28)	Original question
27.b. What information or messages did you receive during your pregnancy about nutrition or diet?<br><br>First allow respondent to provide any answers and check all that apply<br><br>Then probe to identify each response and record all mentioned<br>[ask each answer individually]	Were you told to…<br><br>□ Eat more (quantity)<br>□ Eat a variety of foods rich with iron, vitamin A or C<br>□ Take iron tablets (IFA's)<br>□ Take calcium tablets<br>□ How to manage nausea/vomiting<br>□ Other (specify) ______<br><br>0= No<br>1= Yes, without prompting<br>2= Yes, with prompting<br>9= Don't know	Original question

For peer review only

Supplementary Table 1. List of questions included from the short form Marlowe Crown social desirability index, inclusion in the score

Code	Question	% Yes	Negative key[1]
SM1	Do you sometimes find it hard to go on with your work if you are not encouraged?	39.6	No
SM2	Do you sometimes feel resentful when you don't get your way?	10.0	No
SM3	Do you occasionally give up doing something because you don't think you have the ability	31.1	No
SM4	Are there any times when you felt like rebelling against people in authority even though you knew they were right?	59.9	No
SM5	Are you always a good listener no matter who you are talking to?	21.9	Yes
SM6	Are there any occasions when you took advantage of sometime?	89.1	No
SM7	When you make a mistake are you always willing to admit it?	64.7	Yes
SM8	Are you always courteous, even to people who are disagreeable/not pleasant?	19.5	Yes
SM9	Have you ever been irked when people expressed ideas very different from your own?	14.4	No
SM10	Are there any times when you were quite jealous of the good fortune of others?	85.1	No
SM11	Do you sometimes get irritated/annoyed by people who ask you to do something for them?	27.6	No
SM12	Have you ever deliberately said something that hurt someone's feelings	89.5	No

For peer review only

Supplementary Table 3. Correlations between questions used for assessment of the social desirability

		SM1	SM2	SM3	SM4	SM5	SM6	SM7	SM8	SM9	SM10	SM11	SM12
SM1	Do you sometimes find it hard to go on with your work if you are not encouraged?	1.00											
SM2	Do you sometimes feel resentful when you don't get your way?	0.31 ***	1.00										
SM3	Do you occasionally give up doing something because you don't think you have the ability	0.07	0.08	1.00									
SM4	Are there any times when you felt like rebelling against people in authority even though you knew they were right?	-0.03	0.10 *	-0.08	1.00								
SM5	Are you always a good listener no matter who you are talking to?	0.15 **	0.16 **	0.03	0.22 ***	1.00							
SM6	Are there any occasions when you took advantage of sometime?	.02	.01	-0.01	0.11 *	-0.03	1.00						
SM7	When you make a mistake are you always willing to admit it?	.08	0.04	-0.12 *	0.36 ***	0.23 ***	-0.04	1.00					
SM8	Are you always courteous, even to people who are disagreeable/not pleasant?	.21 ***	0.21 ***	0.03	0.03	0.38 ***	-0.03	0.21 ***	1.00				
SM9	Have you ever been irked when people expressed ideas very different from your own?	.20 ***	0.34 ***	-0.03	0.15 **	0.16 **	0.07	0.13 *	0.32 ***	1.00			
SM10	Are there any times when you were quite jealous of the good fortune of others?	0.16 **	0.07	0.04	0.15 *	0.07	0.06	0.15 **	0.05	0.11 *	1.00		
SM11	Do you sometimes get irritated/annoyed by people who ask you to do something for them?	.29 ***	0.34 ***	0.01	0.15 **	0.28 ***	-0.02	0.21 ***	0.32 ***	0.29 ***	0.15 **	1.00	
SM12	Have you ever deliberately said something that hurt someone's feelings	-0.11 *	-0.02	0.005	0.02	0.07	0.17 **	-0.04	-0.02	-0.07	0.06	0.03	1.00

***p<0.0001, **p<0.01 *p<0.05

For peer review only

Supplementary Table 4. Characteristics of women and households by presence of others during the interview

	No other adults present (n=195)	Any other adult present (n=207)	Husband present (n=28)	Mother in law present (n=98)	Friend present (n=50)	Overall (n=402)
Women's age, years (mean, sd)	22.7 (4.3)	22.3 (4.1)	20.6 (2.9)	21.8 (3.7)	23.4 (4.8)	22.5 (4.2)
# Observed ANC visits	5.0 (2.6)	4.3 (2.3)	4. 1 (2.1)	4.4 (2.5)	4.4 (2.3)	4.7 (2.5)
Socioeconomic status quartile %						
1 (Bottom)	34.9	38.7	32.1	36.7	40.0	36.8
2	18.0	17.4	21.4	17.4	16.0	17.7
3	31.3	31.4	32.1	29.6	38.0	31.3
4 (Top)	15.9	12.6	14.3	16.3	6.0	14.2
Any prior live births (%)	70.3	67.2	60.7	59.2	74.0	68.7
Any formal education (%)	42.1	38.6	42.9	44.9	24.0	40.3
Trimester at enrollment						
1-3 months	46.2	38.7	28.6	40.8	34.0	42.3
4-6 months	51.8	59.9	71.4	57.1	66.0	56.0
7-9 months	2.1	1.5	0	2.0	0	1.7
Got help during interview*	0	52.0	50.0	48.1	32.7	19.9

For peer review only

Supplementary Table 5. Receipt of help during the interview and accuracy of recall of services

	Received help during interview from another adult ARR (95% CI)	Received help from MIL during the interview ARR (95% CI)
Deworming	0.97 (0.78, 1.20)	0.94 (0.78, 1.13)
Receipt of Ca or Ca info	0.99 (0.82, 1.20)	1.01 (0.90, 1.13)
Counseling on		
Nutrition	0.97 (0.85, 1.11)	No convergence
Eating more	0.93 (0.77, 1.12)	No convergence
Diverse diet	No convergence	No convergence
Managing nausea	1.02 (0.70, 1.49)	1.18 (0.91, 1.53)
Not drinking *paan* alcohol	0.94 (0.64, 1.38)	1.24 (1.00, 1.55)
Not smoking/ using	0.88 (0.60, 1.29)	1.14 (0.92, 1.41)
Accuracy of IFA estimate		
Within 30 tablets	1.08 (0.69, 1.69)	1.16 (0.81, 1.64)
Within 60 tablets	1.04 (0.80, 1.36)	1.07 (0.85, 1.34)
Within 90 tablets	1.05 (0.89, 1.24)	1.09 (0.96, 1.25)
Estimate number of ANC visits		
Exactly	0.47 (0.16, 1.38)	0.76 (0.39, 1.49)
Within 1 visit	0.86 (0.57, 1.29)	0.81 (0.58, 1.13)
Within 2 visits	0.98 (0.78, 1.23)	1.02 (0.86, 1.21)

For peer review only

STROBE Statement—checklist of items that should be included in reports of observational studies

	Item No	Recommendation	Page No
**Title and abstract**	1	(*a*) Indicate the study's design with a commonly used term in the title or the abstract	2
		(*b*) Provide in the abstract an informative and balanced summary of what was done and what was found	2
**Introduction**			
Background/rationale	2	Explain the scientific background and rationale for the investigation being reported	4
Objectives	3	State specific objectives, including any prespecified hypotheses	5
**Methods**			
Study design	4	Present key elements of study design early in the paper	6-7
Setting	5	Describe the setting, locations, and relevant dates, including periods of recruitment, exposure, follow-up, and data collection	6
Participants	6	(*a*) *Cohort study*—Give the eligibility criteria, and the sources and methods of selection of participants. Describe methods of follow-up<br>*Case-control study*—Give the eligibility criteria, and the sources and methods of case ascertainment and control selection. Give the rationale for the choice of cases and controls<br>*Cross-sectional study*—Give the eligibility criteria, and the sources and methods of selection of participants	8
		(*b*) *Cohort study*—For matched studies, give matching criteria and number of exposed and unexposed<br>*Case-control study*—For matched studies, give matching criteria and the number of controls per case	
Variables	7	Clearly define all outcomes, exposures, predictors, potential confounders, and effect modifiers. Give diagnostic criteria, if applicable	6-9
Data sources/ measurement	8*	For each variable of interest, give sources of data and details of methods of assessment (measurement). Describe comparability of assessment methods if there is more than one group	6-8
Bias	9	Describe any efforts to address potential sources of bias	Entire paper
Study size	10	Explain how the study size was arrived at	7
Quantitative variables	11	Explain how quantitative variables were handled in the analyses. If applicable, describe which groupings were chosen and why	9
Statistical methods	12	(*a*) Describe all statistical methods, including those used to control for confounding	9
		(*b*) Describe any methods used to examine subgroups and interactions	na
		(*c*) Explain how missing data were addressed	9
		(*d*) *Cohort study*—If applicable, explain how loss to follow-up was addressed<br>*Case-control study*—If applicable, explain how matching of cases and controls was addressed<br>*Cross-sectional study*—If applicable, describe analytical methods taking account of sampling strategy	9, Supp fig 1

For peer review only - http://bmjopen.bmj.com/site/about/guidelines.xhtml

(*e*) Describe any sensitivity analyses

Continued on next page

For peer review only

**Results**			
Participants	13*	(a) Report numbers of individuals at each stage of study—eg numbers potentially eligible, examined for eligibility, confirmed eligible, included in the study, completing follow-up, and analysed	Sup Fig 1, 10
		(b) Give reasons for non-participation at each stage	Supp fig 1
		(c) Consider use of a flow diagram	Supp fig 1
Descriptive data	14*	(a) Give characteristics of study participants (eg demographic, clinical, social) and information on exposures and potential confounders	Table 1; supp table 4
		(b) Indicate number of participants with missing data for each variable of interest	tables
		(c) *Cohort study*—Summarise follow-up time (eg, average and total amount)	Page 8
Outcome data	15*	*Cohort study*—Report numbers of outcome events or summary measures over time	*tables*
		Case-control study—Report numbers in each exposure category, or summary measures of exposure	
		Cross-sectional study—Report numbers of outcome events or summary measures	
Main results	16	(*a*) Give unadjusted estimates and, if applicable, confounder-adjusted estimates and their precision (eg, 95% confidence interval). Make clear which confounders were adjusted for and why they were included	No room for unadjusted.
		(*b*) Report category boundaries when continuous variables were categorized	9
		(*c*) If relevant, consider translating estimates of relative risk into absolute risk for a meaningful time period	na
Other analyses	17	Report other analyses done—eg analyses of subgroups and interactions, and sensitivity analyses	Supp tables
**Discussion**			
Key results	18	Summarise key results with reference to study objectives	10-11
Limitations	19	Discuss limitations of the study, taking into account sources of potential bias or imprecision. Discuss both direction and magnitude of any potential bias	13-14
Interpretation	20	Give a cautious overall interpretation of results considering objectives, limitations, multiplicity of analyses, results from similar studies, and other relevant evidence	13-14,
Generalisability	21	Discuss the generalisability (external validity) of the study results	14
**Other information**			
Funding	22	Give the source of funding and the role of the funders for the present study and, if applicable, for the original study on which the present article is based	1

*Give information separately for cases and controls in case-control studies and, if applicable, for exposed and unexposed groups in cohort and cross-sectional studies.

Note: An Explanation and Elaboration article discusses each checklist item and gives methodological background and published examples of transparent reporting. The STROBE checklist is best used in conjunction with this article (freely available on the Web sites of PLoS Medicine at http://www.plosmedicine.org/, Annals of Internal Medicine at http://www.annals.org/, and Epidemiology at http://www.epidem.com/). Information on the STROBE Initiative is available at www.strobe-statement.org.

For peer review only - http://bmjopen.bmj.com/site/about/guidelines.xhtml

BMJ Open

How does social desirability bias influence survey-based estimates of the use of antenatal care in rural Nepal?

Journal:	*BMJ Open*
Manuscript ID	bmjopen-2022-071511.R1
Article Type:	Original research
Date Submitted by the Author:	18-Jun-2023
Complete List of Authors:	Thorne-Lyman, Andrew; Johns Hopkins University Bloomberg School of Public Health, International Health<br>Lama, Tsering; Johns Hopkins University Bloomberg School of Public Health, International Health; Nepal Nutrition Intervention Project, Sarlahi<br>Heidkamp, Rebecca; Johns Hopkins University Bloomberg School of Public Health, International Health<br>Munos, Melinda; Johns Hopkins University, Bloomberg School of Public Health, Department of International Health<br>Manandhar, Porcia; Johns Hopkins University Bloomberg School of Public Health, International Health<br>Khatry, Subarna; Nepal Nutr Intervent Project Sarlahi; Johns Hopkins University Bloomberg School of Public Health, International Health<br>Bryce, Emily; Johns Hopkins University Bloomberg School of Public Health, International Health<br>LeClerq, Steven C.; Johns Hopkins Bloomberg School of Public Health, Department of International Health; Nepal Nutrition Intervention Project<br>Katz, Joanne; Johns Hopkins University Bloomberg School of Public Health, International Health
**Primary Subject Heading**:	Global health
Secondary Subject Heading:	Health services research, Nutrition and metabolism, Public health, Research methods
Keywords:	Nutrition < TROPICAL MEDICINE, PRIMARY CARE, PUBLIC HEALTH, OBSTETRICS

SCHOLARONE™
Manuscripts

I, the Submitting Author has the right to grant and does grant on behalf of all authors of the Work (as defined in the below author licence), an exclusive licence and/or a non-exclusive licence for contributions from authors who are: i) UK Crown employees; ii) where BMJ has agreed a CC-BY licence shall apply, and/or iii) in accordance with the terms applicable for US Federal Government officers or employees acting as part of their official duties; on a worldwide, perpetual, irrevocable, royalty-free basis to BMJ Publishing Group Ltd ("BMJ") its licensees and where the relevant Journal is co-owned by BMJ to the co-owners of the Journal, to publish the Work in this journal and any other BMJ products and to exploit all rights, as set out in our licence.

The Submitting Author accepts and understands that any supply made under these terms is made by BMJ to the Submitting Author unless you are acting as an employee on behalf of your employer or a postgraduate student of an affiliated institution which is paying any applicable article publishing charge ("APC") for Open Access articles. Where the Submitting Author wishes to make the Work available on an Open Access basis (and intends to pay the relevant APC), the terms of reuse of such Open Access shall be governed by a Creative Commons licence – details of these licences and which Creative Commons licence will apply to this Work are set out in our licence referred to above.

Other than as permitted in any relevant BMJ Author's Self Archiving Policies, I confirm this Work has not been accepted for publication elsewhere, is not being considered for publication elsewhere and does not duplicate material already published. I confirm all authors consent to publication of this Work and authorise the granting of this licence.

For peer review only

Title: How does social desirability bias influence survey-based estimates of the use of antenatal care in rural Nepal?

Authors: Andrew L. Thorne-Lyman[a], Tsering P. Lama[a,b], Rebecca A. Heidkamp[a], Melinda K. Munos[a], Porcia Manandhar[a], Subarna K. Khatry[a,b], Emily Bryce[a] Steven C. LeClerq[a,b], Joanne Katz[a]

[a]Department of International Health, Johns Hopkins Bloomberg School of Public Health, Baltimore, MD, 21205, USA

[b]Nepal Nutrition Intervention Project-Sarlahi, Kathmandu, Nepal

Corresponding author: Andrew L. Thorne-Lyman, Program in Human Nutrition, Department of International Health, 615 N. Wolfe Street E2545, Johns Hopkins Bloomberg School of Public Health, Baltimore, Maryland, USA. **Email:** athorne1@jhu.edu, **Phone**: +1 856 214-5755, **Fax**:+1 410-955-0196

Sources of support: Bill & Melinda Gates Foundation INV-007332 and OPP1172551

Competing interests: None

Keywords: Nepal, survey, nutrition, social desirability bias, antenatal care, recall, validity

For peer review only

For peer review only - http://bmjopen.bmj.com/site/about/guidelines.xhtml

Abstract

Introduction

Large scale surveys are used to assess the coverage of antenatal care (ANC) and nutrition interventions. Social desirability bias could affect the validity of questions about the receipt of care and coverage estimates, though this has not been well studied.

Methods

A longitudinal cohort design was used comparing direct observation of receipt of ANC and 6 specific nutrition components of ANC including different counselling and iron-folic acid against reports of receipt using a post-partum survey (n=401). The presence of socially desirable responses was assessed using a short form Marlowe-Crowne social desirability index and the presence of other interview observers as a separate exposure. Log-binomial multivariable regression was used to predict accuracy and overestimation of recalled receipt of ANC services against observed receipt adjusting for age, education, first-pregnancy, and socio-economic status.

Results

The social desirability score did not significantly predict accuracy or overestimation of most types of ANC care except counselling on nausea: higher social desirability scores were associated with more accurate recall [adjusted RR, aRR 1.08 (95% CI 1.03, 1.12)] and less overestimation, [aRR 0.85 (0.80, 0.91)]. Mothers' interviews with their mothers-in-law or husbands present were more likely to overestimate the number of ANC visits they received by more than 3 visits [aRR 2.07 (1.11, 3.84)] and [aRR 4.19 (2.17, 8.10)] respectively. Those interviewed with friends present tended to overestimate the receipt of counselling on nausea, avoiding alcohol, and not smoking.

Conclusion

For peer review only - http://bmjopen.bmj.com/site/about/guidelines.xhtml

Findings suggest that the presence of observers can lead to overestimation of the receipt of ANC care and support efforts to interview in private settings. Findings that the social desirability score did not predict the accuracy of most types of ANC care might reflect a reality that such questions may not be sensitive from a social-norms perspective and additional local adaptation of the tool may be useful.

Strengths and limitations of the study

- The effect of social desirability bias and presence of interview observers on response accuracy was evaluated against the gold standard of observed receipt of antenatal care services.
- The tool used to assess social desirability was adapted for comprehension for the local population in rural Nepal, but has not been validated for that context.
- Interviewers noted the presence of others at any point during the interview, and not specifically during specific portions of the interview, likely attenuating estimates of the relationship between presence of others and validity of reported services receipt.

For peer review only

Introduction

The World Health Organization (WHO) recommends a number of nutrition interventions in pregnancy, including iron-folic acid supplements (IFA), counselling on healthy eating and physical activity, and in certain contexts calcium, micronutrient supplements and balanced energy and protein supplements.[1] Large-scale household surveys, including those implemented by the Demographic Health Survey program, are used across low and middle-income countries to measure coverage of antenatal interventions in order to monitor progress towards targets and evaluate policies and programs.[2]

Many survey-based coverage indicators come from self-reported data and a growing number of studies have explored the validity of responses to questions about the receipt of nutrition interventions delivered through antenatal care.[2][3] A study carried out by our research team in Nepal found that respondents tend to overestimate the receipt of IFA in pregnancy, and that overestimation was not explained by either maternal or household characteristics.[4] Women's reports of the receipt of various counselling interventions, weight measurement, and deworming were generally recalled more accurately than questions about other services such as counselling to not drink alcohol or tobacco.[5]

Social desirability response bias (SDB) is a common type of bias that reflects the tendency of respondents to reply in a manner that may be viewed positively by social peers or that is consistent with social norms and expectations. Social desirability bias (SDB) is generally thought to be greater for questions that are perceived as sensitive or subject to judgment such as those related to sexual practices or family planning.[6,7] The perceived relationships between respondents and interviewers or observers can also influence socially desirable responses. For example, a study administered in 14 African countries found that respondents give systematically different answers to interviewers depending on whether or not they were from their same ethnic group.[8]

For peer review only - http://bmjopen.bmj.com/site/about/guidelines.xhtml

Survey-based scales to measure social desirability were developed in the field of social psychology in the 1960's beginning with the 33-question Marlowe-Crowne scale[9]. Shorter versions of these scales have been shown to be reliable across different cultural contexts, though more work is needed to refine and test them.[7,10, 11]. These scales include a variety of positively keyed questions that are "culturally acceptable but improbable" or "desirable but rare" and negatively keyed items that are "culturally unacceptable but probable" or "undesirable but common".[10] The scales are used to detect and quantify the extent of socially desirable responses in surveys. Where it has been detected, a number of approaches exist correct for it or mitigate its effects including a posteriori statistical adjustment for SDS,[12,13] excluding high SDS scorers[13] or rephrasing questions using forgiving wording[14].

Social desirability bias has been frequently described as an area of potential concern in nutrition-related studies collecting data about diet, physical activity, and self-reported weight, but few studies have directly quantified the strength of association between social desirability scores and these domains.[15] Evaluations of the Alive & Thrive infant and young child feeding program in Vietnam and Bangladesh explored whether adjusting for short-form social desirability scales affected estimates of program impact on diet, but found that results were unchanged.[16,17] To our knowledge, no previous studies have directly explored whether SDB or presence of others during survey interviews is a potential explanation for inaccurate survey responses about the receipt of ANC and related nutrition interventions. The aims of this study were therefore to:

1. Adapt and test a modified short-form version of the Marlow-Crowne social desirability scale in rural Nepal.
2. Explore whether social desirability or the presence of other adults during interviews helps to explain previous findings related to overestimation of the receipt of services.

For peer review only - http://bmjopen.bmj.com/site/about/guidelines.xhtml

3. Explore whether social desirability or the presence of other adults during interviews is associated with inaccurate recall of ANC and associated services.

Methods

Study area and population

This study took place in Sarlahi District, an area in the southern plains (*Tarai)* of Nepal located in Province 2, the province with the lowest rates of ANC coverage in Nepal. According to the 2016 Demographic and Health Survey, only 36% of women in Province 2 received all four recommended ANC visits, far below the national average of 59%.[18]. These low rates appear paradoxical given that 70% of the population in Sarlahi lived within 30 minutes of a government health facility. This paradox may be explained in part by low rates of maternal education and gender norms that limit women's ability to travel outside of the household.[19] The population of Sarlahi is linguistically and culturally heterogeneous, including both Nepali and Maithili-speaking groups.[20]

Antenatal care and nutrition services delivered through ANC

At the time of our study, Nepal was implementing the World Health Organization's focused ANC (FANC) model, with recommended visits occurring in the 4th, 6th, $8^{th,}$ and 9th months of pregnancy. The government incentivizes ANC participation by providing 800 rupees (~7 USD) to women who complete all four visits.[21] Nutrition interventions delivered through ANC in Nepal include the provision of iron-folic acid supplements, checking of weight, and counselling on various topics related to nutrition and breastfeeding.

Study design

This study was part of a larger effort to validate coverage indicators for various nutrition services through ANC, described in detail elsewhere.[4,5] In brief, study staff were based at five government

For peer review only - http://bmjopen.bmj.com/site/about/guidelines.xhtml

health posts and trained to directly observe and record the receipt of ANC services by enrolled women. The observers' 28-item checklist included details about the provision of iron-folic acid pills (e.g. how many pills women received, whether they were instructed to purchase iron folic acid tablets or syrup), measurement of weight, and types of counselling on nutrition and breastfeeding. These direct observations served as a "gold standard" to validate maternal recall of ANC services received at study clinics. Maternal recall was assessed by interviewing enrolled women using a structured questionnaire around six months after delivery. [2,4,5] The interview questions were drawn from the official translated Nepali DHS 2016 questionnaire (DHS Round 7), a draft of the DHS Round 8 questionnaire, and novel questions to assess different components of counselling and weight gain assessment (**Supplementary Table 1)**. The six-month visits were conducted at the women's homes or their parental homes. Women were asked about socioeconomic status at enrollment to the validation study and during the visit at six months after delivery. As described in detail elsewhere [4], a target sample size of 300 women was established for the study based on an assumption of 50% coverage for receipt of IFA and lower coverage for counselling. After accounting for loss-to-follow-up, the possibility of women going elsewhere for ANC, and of non-live birth outcomes, a target study enrollment of 450 women was established.

Measurement of social desirability

Questions from a short-form Marlowe-Crowne social desirability index (**Supplementary Table 2)** were translated and back-translated by Nepali/English speakers familiar with the study context, pre-tested and slightly adapted to the local context. During pre-testing one of the questions was not well understood and dropped; 12 questions were included in the six-month visit questionnaire.[11] Study staff also recorded whether any other adult had been present at any point during the interview (with specific codes for husbands, mothers-in law, friends of the respondent or other adults), as well as whether those present had helped the respondent to answer any questions.

For peer review only - http://bmjopen.bmj.com/site/about/guidelines.xhtml

Correlations between different items of the social desirability index were explored after reverse coding negatively keyed questions, and most were found to be positively associated with one another (**Supplementary Table 3**). Tests of internal consistency revealed the full 12-item scale to have a Cronbach's alpha of 0.58 and only minor improvements (to an alpha of 0.65) were possible by removing several items so the full 12-item scale was used in analyses. On visual inspection, scores on the social desirability scale (SDS) were only slightly skewed, with a mean of 5.5 and a standard deviation of 2.1.

Data collection

Enrollment criteria for the parent trial included currently pregnant, married women aged 15 years or older, who a) resided in the study area, b) visited one of the five health posts for their first ANC visit and c) had the intention to return to these study sites for subsequent ANC visits. All interview questions were translated and back-translated by Nepali/English speakers familiar with the study context. These questions were pre-tested and minor changes were made to help facilitate comprehension in the local context. Both Maithili and Nepali-speaking interviewers conducted the interviews following training exercises designed to standardize the approach to translation. Interviewers were trained to replicate usual survey conditions. This included asking respondents to answer questions themselves even if a family member had tried to answer it for her and repeating or rephrasing questions in a simpler way if respondents were confused or responded with "I don't know".

The study started in December 2018 and reached full enrollment by November 2019. Direct observations of ANC visits were nearly completed by March 2020 when a shut-down for the Covid-19 pandemic interrupted all non-emergency health services in Nepal. Data collection for the post-partum six-month visit was started in September 2019 but Covid-19 related shutdowns caused delays. The mean number of days that passed from delivery to the post-partum data collection visit was 252 with a standard deviation of 75 days. Precautions taken to prevent the spread of Covid-19 included masking by

For peer review only

For peer review only - http://bmjopen.bmj.com/site/about/guidelines.xhtml

167 interviewers and respondents, handwashing with soap provided to interviewees, holding interviews
168 outdoors when weather permitted, and maintaining a distance of more than six feet during data
169 collection.

170 *Data analysis*

171 We examined whether SDS or presence of other adults during interviews was associated with the
172 accuracy or overestimation of recalled receipt of specific ANC services using log-binomial bivariate and
173 multivariable regressions to directly estimate risk ratios or log-Poisson models in the case of model non-
174 convergence [22]. Multivariable analyses adjusted for age (continuous), education (any vs. none), first
175 pregnancy (yes/no), and quartile of socioeconomic status as possible confounders. We used a theory-
176 based approach to selection of covariates for these models. For interpretation purposes, high SDS
177 corresponds to women that would be more likely to be exhibit socially desirable responses. SDS was
178 treated as both a continuous score and as a dichotomous variable in analyses, with the upper quartile
179 defined as those with a score of 7 or greater compared against the lower three quartiles, recognizing
180 that socially desirable response bias may not be a linear construct. For receipt of counselling
181 interventions, calcium, and deworming, accurate recall was defined as alignment (yes/no) of the follow-
182 up interview responses with observed receipt or non-receipt of those interventions.[4,5] Overestimation
183 for these interventions was defined as respondents that reported receipt but were not observed
184 receiving it, and for each intervention women were categorized as over- estimators or non-over-
185 estimators. For ANC visits and IFA, several different thresholds were used to define accuracy and the
186 degree of overestimation. For reasons of statistical power, we restricted our analysis to questions about
187 interventions with directly observed coverage below 90%. A complete case analysis was used for the
188 treatment of missing data and loss to follow-up was minimal (**Supplementary Figure 1**).

189 *IRB approval*

For peer review only - http://bmjopen.bmj.com/site/about/guidelines.xhtml

190 This research conformed with the principles embodied in the Declaration of Helsinki. The institutional review boards at Johns Hopkins Bloomberg School of Public Health and the Nepal Health Research Council both approved this study including permission to collect and resume data collection during the Covid-19 epidemic. Signed consent was obtained from all participants. Patients and the public were not involved in the design, conduct, reporting or dissemination plans of this research. Dissemination and discussion of study design and results is made to a national committee and a local district committee.

Patient and public involvement

The research questions were not informed through direct consultation of patients' priorities, experience or preferences. However, many members of our local research team are from the local population where the research is conducted and were involved in pretesting, questionnaire development and recruitment. Findings will be disseminated to national and local stakeholders through meetings and local presentations.

Results

Of the 441 women enrolled in the study, 396 were included in our analysis after accounting for exclusion criteria described in **Supplementary Figure 1**. The mean age, number of observed ANC visits, and birth history were similar among women in the upper quartile of SDS compared those in the lower three quartiles (**Table 1)**. Women with lower SDS tended to have lower education and enrolled in the study later in pregnancy than those with higher scores. Twice as many women with lower SDS had another adult present during the interview than those with higher scores; mothers-in-law and other adults were the most common observers. Those with lower SDS were only slightly more likely to have an observer help them respond to interview questions. Characteristics of women disaggregated by the presence of observers are presented in **Supplementary Table 4**; those with a friend present were less likely to be in the top quartile of socioeconomic status or to have education.

For peer review only - http://bmjopen.bmj.com/site/about/guidelines.xhtml

Table 1. Characteristics of the study population

	Bottom 3 quartiles SDS (n=284)	Upper quartile SDS (n=112)	Overall (n=396)
Age, years, mean (sd)	22.7 (4.3)	21.9 (4.0)	22.5 (4.2)
# Observed ANC visits, mean (sd)	4.6 (2.4)	4.8 (2.6)	4.7 (2.5)
Socioeconomic status quartile (%)			
1 (Bottom)	36.6	39.3	37.4
2	17.6	17.0	17.4
3	31.7	29.5	31.1
4 (Top)	14.1	14.3	14.1
Any prior live births (%)	71.7	71.8	71.7
Any formal education (%)	32.2	22.3	28.3
Trimester at enrollment (%)			
1-3 months	39.4	50.0	42.4
4-6 months	59.2	47.3	55.8
7-9 months	1.4	2.7	1.8
Interview observed by another adult (%)	60.9	31.1	52.1
Husband	7.4	5.9	7.0
Mother-in-law	28.3	14.3	24.4
Friend	15.9	5.0	12.9
Other adult	39.5	16.0	32.9
Observer helped respondent answer questions (%)	29.0	23.5	27.9

About half of women overestimated the number of IFA tablets they received by at least 30 tablets and over a third overestimated the number of ANC visits by at least one visit **(Table 2)**. About a third of women reported receiving counselling for nausea, alcohol or smoking that had not been observed at clinics. Null relations were apparent between SDS and overestimation of receipt of most services, although some associations neared statistical significance when SDS was treated as a dichotomous predictor. Additionally, the dichotomous SDS had a stronger relation with extreme overestimation of reported receipt of both ANC and IFA compared to more modest overestimation. SDS were generally not associated with accuracy of recalled services (**Table 3**). The only exception was for counselling on the management of nausea: women with higher SDS tended to have more accurate recall.

For peer review only

225 Table 2. High social desirability score as a predictor of overestimation of nutrition/ANC services receipt
226 in pregnancy

	N	Proportion Overestimating %	SDS[2] as a predictor of overestimation	
			Dichotomous SDS adjusted[1,3] RR (95% CI)	Continuous SDS adjusted RR[1,3] (95% CI)
Deworming	395	15.2	1.12 (0.67, 1.87)	1.01 (0.90, 1.13)
Receipt or prescribe Ca	390	7.6	0.76 (0.33, 1.75)	0.90 (0.75, 1.07)
Counselling on				
Managing nausea	391	38.3	0.49 (0.34, 0.72)	0.85 (0.80, 0.91)
Not drinking alcohol	395	37.7	1.26 (0.97, 1.63)	1.04 (0.98, 1.10)
Not smoking/ using *paan*	395	31.2	1.23 (0.91, 1.66)	1.02 (0.95, 1.09)
Overestimate IFA receipt				
By >30 tablets	404	51.7	1.07 (0.87, 1.30)	1.02 (0.97, 1.06)
By >60 tablets	404	34.0	1.16 (0.87, 1.54)	1.03 (0.97, 1.10)
By >90 tablets	404	17.5	1.28 (0.83, 1.98)	1.02 (0.93, 1.13)
Overestimate # of ANC visits				
By >1 visit	402	34.8	0.99 (0.74, 1.34)	1.00 (0.94, 1.07)
By >2 visits	402	18.7	1.17 (0.75, 1.82)	1.02 (0.92, 1.13)
By >3 visits	402	9.2	1.37 (0.71, 2.62)	1.02 (0.87, 1.18)

227 [1]Adjusted risk ratio > 1 indicates greater risk of overestimating receipt of services

228 [2]SDS=Social desirability score, treated as upper quartile vs lower three quartiles

229 [3]Adjusted for woman's age (continuous), any education, first pregnancy, and socio-economic status
230 quartile

231 Table 3. High social desirability score as a predictor of accurate recall of nutrition/ANC services receipt in
232 pregnancy

	N	Proportion accurate %	SDS[2] as a predictor of accurate recall	
			Dichotomous SDS adjusted[1,3] RR (95% CI)	Continuous SDS adjusted RR[1,3] (95% CI)
Deworming	395	77.5	0.97 (0.86, 1.09)	0.99 (0.96, 1.01)
Receipt or prescribe Ca	390	85.6	0.98 (0.89, 1.09)	0.99 (0.97, 1.01)
Counselling on				
Managing nausea	391	50.9	1.30 (1.07, 1.60)	1.08 (1.03, 1.12)
Not drinking alcohol	395	55.7	0.89 (0.72, 1.10)	0.97 (0.93, 1.02)
Not smoking/ using *paan*	395	59.5	0.91 (0.74, 1.11)	0.98 (0.94, 1.02)
Overestimate IFA receipt				
By >30 tablets	396	39.9	0.87 (0.66, 1.16)	0.98 (0.93, 1.04)
By >60 tablets	396	61.6	1.01 (0.85, 1.20)	1.02 (0.98, 1.05)
By >90 tablets	396	79.3	0.91 (0.80, 1.03)	1.00 (0.97, 1.02)
Overestimate # of ANC visits				
By >1 visit	396	21.0	0.94 (0.60, 1.45)	1.01 (0.92, 1.11)
By >2 visits	396	55.1	0.91 (0.74, 1.12)	1.02 (0.97, 1.06)
By >3 visits	396	75.5	0.96 (0.84, 1.10)	1.01 (0.98, 1.04)

For peer review only - http://bmjopen.bmj.com/site/about/guidelines.xhtml

For peer review only

[1]Adjusted risk ratio > 1 indicates more accurate recall

[2]SDS=Social desirability score, treated as upper quartile vs lower three quartiles

[3]Adjusted for woman's age (continuous), any education, first pregnancy, and socio-economic status quartile

The presence of any other adult was significantly associated with overestimation of receipt of counselling on managing nausea as well as overestimation of the number of ANC visits. Mothers-in-law (MILs) were present during about a quarter of all interviews. Their presence was associated with significant overestimation of the receipt of deworming tablets [ARR 1.90 (95% C.I. 1.18, 3.05)] and more than doubled the risk of overestimating the number of ANC visits by 3 or more visits [ARR 2.07 (95% 1.11, 3.84)] **(Table 4)**. Interestingly, the presence of a MIL during the interview appeared to attenuate overestimation of IFA receipt by >90 tablets, though this estimate included the null (ARR: 0.56 (95% CI 0.31, 1.01). The presence of a husband was less common (7%) and was significantly associated with the overestimation of ANC visits by one, two and three visits. The presence of a friend was significantly associated with overestimation of the receipt of multiple types of ANC services including information about calcium, managing nausea, not drinking alcohol and not smoking or using tobacco.

Table 4**.** Presence of others during the interview as a predictor of overestimation of recalled services receipt

	N	Mother in law present ARR[2] (95% CI)	Husband present ARR[2] (95% CI)	Friend present ARR[2] (95% CI)	Any adult present ARR[2] (95% CI)
Deworming	401	1.90 (1.18, 3.05)	0.50 (0.13, 1.94)	0.85 (0.41, 1.76)	1.05 (0.66, 1.67)
Receipt or prescribe Ca	396	1.02 (0.45, 2.32)	1.63 (0.52, 5.06)	2.69 (1.30, 5.55)	1.91 (0.92, 3.97)
Counselling on					
Managing nausea	397	1.17 (0.89, 1.54)	1.09 (0.68, 1.76)	1.53 (1.15, 2.04)	1.70 (1.30, 2.22)
Not drinking alcohol	401	0.80 (0.58, 1.11)	1.07 (0.66, 1.74)	1.41 (1.05, 1.90)	1.09 (0.85, 1.39)
Not smoking/ using paan	401	0.71 (0.48, 1.06)	1.22 (0.73, 2.06)	1.55 (1.12, 2.15)	0.98 (0.74, 1.31)
Overestimate IFA receipt					
By >30 tablets	402	0.94 (0.76, 1.17)	0.88 (0.60, 1.29)	0.93 (0.69, 1.25)	0.96 (0.80, 1.15)
By >60 tablets	402	0.86 (0.62, 1.19)	1.13 (0.72, 1.77)	0.89 (0.58, 1.37)	0.95 (0.73, 1.24)
By >90 tablets	402	0.56 (0.31, 1.01)	0.90 (0.40, 2.02)	0.96 (0.51, 1.81)	0.73 (0.49, 1.11)
Overestimate # of ANC visits					

For peer review only

For peer review only - http://bmjopen.bmj.com/site/about/guidelines.xhtml

By >1 visit	400	1.28 (0.97, 1.70)	1.48 (1.03, 2.13)	1.18 (0.82, 1.70)	1.33 (1.01, 1.73)
By >2 visits	400	1.32 (0.85, 2.05)	1.97 (1.13, 3.44)	1.24 (0.70, 2.20)	1.57 (1.03, 2.40)
By >3 visits	400	2.07 (1.11, 3.84)	4.19 (2.17, 8.10)	0.43 (0.10, 1.72)	2.26 (1.15, 4.46)

[1]Adjusted risk ratio > 1 indicates greater risk of overestimation of reported services receipt

[3]Adjusted for woman's age (continuous), any education, first pregnancy, and socio-economic status quartile

Women interviewed in the presence of MILs were less accurate in estimating the number of ANC visits but more accurate in recalling the receipt of counselling related to avoiding alcohol consumption in pregnancy. Women tended to be less accurate when estimating the number of ANC visits or receipt of IFA in front of their husbands. (**Table 5**).

Table 5 Presence of others during the interview as a predictor of accuracy of recalled services receipt

	**N**	**Mother in law present RR (95% CI)**	**Husband present RR (95% CI)**	**Friend present RR (95% CI)**	**Any adult present RR (95% CI)**
Deworming	401	0.91 (0.79, 1.04)	1.13 (0.99, 1.29)	0.84 (0.57, 1.25)	1.03 (0.93, 1.14)
Receipt or prescribe Ca	396	1.03 (0.94, 1.12)	0.97 (0.81, 1.16)	0.87 (0.74, 1.04)	0.99 (0.92, 1.08)
Counselling on					
Managing nausea	397	0.94 (0.74, 1.19)	0.78 (0.49, 1.25)	0.71 (0.50, 1.02)	0.72 (0.59, 0.87)
Not drinking alcohol	401	1.20 (1.00, 1.45)	1.07 (0.79, 1.45)	0.78 (0.55, 1.10)	1.01 (0.85, 1.20)
Not smoking/ using paan	401	1.14 (0.95, 1.35)	0.91 (0.66, 1.27)	0.76 (0.55, 1.05)	1.04 (0.89, 1.22)
Accuracy of IFA estimate					
Within 30 tablets	402	0.96 (0.73, 1.28)	1.06 (0.68, 1.68)	1.17 (0.84, 1.63)	1.01 (0.80, 1.29)
Within 60 tablets	402	0.91 (0.75, 1.11)	0.76 (0.50, 1.14)	0.96 (0.76, 1.20)	0.94 (0.81, 1.09)
Within 90 tablets	402	1.00 (0.89, 1.12)	0.69 (0.50, 0.96)	0.96 (0.82, 1.13)	0.99 (0.90, 1.10)
Estimate # of ANC visits					
Exactly	400	0.67 (0.41, 1.10)	0.47 (0.16, 1.40)	1.25 (0.75, 2.08)	0.94 (0.65, 1.36)
Within 1 visit	400	0.86 (0.69, 1.08)	0.87 (0.59, 1.27)	1.07 (0.83, 1.37)	1.04 (0.87, 1.24)
Within 2 visits	400	0.85 (0.74, 0.99)	0.76 (0.56, 1.04)	1.07 (0.92, 1.24)	0.93 (0.82, 1.04)

[1]Adjusted risk ratio > 1 indicates greater accuracy of reported services receipt

[3]Adjusted for woman's age (continuous), any education, first pregnancy, and socio-economic status quartile

We also examined how the receipt of help from observers to answer questions during the interview may have affected the accuracy of recall of services **(Supplementary Table 5)**. Assistance from MIL was

For peer review only

For peer review only - http://bmjopen.bmj.com/site/about/guidelines.xhtml

associated with more accurate recall of counselling to not drink alcohol [ARR 1.24 (95% C.I. 1.00, 1.55) but no other services. In contrast, assistance from husbands did not significantly affect the accuracy of recall of any services.

Discussion

Previous studies exploring the validity of self-reported questions about the receipt of ANC care have raised social desirability bias as a possible type of error that might affect coverage estimates.[23,24] To our knowledge, however, this is the first study in the peer-reviewed literature to directly explore whether social desirability bias or the presence of others influences the validity of women's recalled receipt of ANC services. We found that SDS did not significantly predict the accuracy of women's recall of most types of ANC services, except for counselling on the management of nausea. However, the presence of another adult observer during the interview did influence the accuracy of recall. Women that were interviewed with their MIL or husbands present tended to overestimate the number of ANC visits they had received and those interviewed with friends present tended to overestimate the receipt of different types of counselling.

For peer review only

The effects of social desirability bias are complex[25] and socially desirable responses may be driven by either unconscious distortions related to self-deception or impression management related to social norms.[13,26] The potential for social desirability bias to affect questions about nutrition and receipt of ANC services in low- and middle-income countries has only been indirectly explored in the literature. For example, a study conducted in China found that recalled receipt of Hepatitis B blood tests (HBsAb) was much higher than HIV tests despite similar observed coverage and suggested that the difference might be explained by socially desirable responses.[23] Many of the questions that we tested in our study related to whether or not women had received specific nutrition interventions as part of

For peer review only - http://bmjopen.bmj.com/site/about/guidelines.xhtml

ANC visits. A likely explanation for null associations with the SDS is that such questions do not reflect practices that might be perceived negatively (or positively) from the standpoint of social norms.[25]

However, it is unclear why higher scores on the SDS were positively associated with more accurate recall of counselling on management of nausea. One possible explanation is that the counselling focused only on women that complained of nausea and not all women.[5] Thus, it is possible that the socially desirable response to this question was to accurately respond to whether or not counselling had been received, and that these responses were particularly easy to recall because they were related to recollections of nausea.

In contrast to questions about the simple receipt of services during ANC visits, questions about the number of ANC visits or IFA consumption could be more subject to social desirability bias because they relate to a woman's initiative in seeking services or taking supplements and because there is likely positive sentiment around the benefits of services for women and their babies. This could explain the finding that the presence of MILs and/or husbands during the interview was associated with a significantly greater overestimation of the number of ANC visits. Cognitive testing[27] conducted in this same population revealed that women were confused about whether visits to providers outside of health posts including for sonograms should be counted towards their estimate of ANC visits or not, and it is possible that women might be more likely to count such visits when in the presence of other household members.

IFA coverage is an important indicator for global monitoring of nutrition interventions; it is included in the Global Nutrition Monitoring Framework and an indicator for the Countdown to 2030.[28,29] There are global concerns about accuracy of recall of the number of tablets received or consumed during a specific pregnancy, particularly in the DHS or other surveys where respondents may be 2-5 years postpartum. Previous work in Nepal showed that at six months post-partum women's self-

For peer review only

For peer review only - http://bmjopen.bmj.com/site/about/guidelines.xhtml

reported receipt of IFA tended to overestimate the number of supplements that they actually received and that this overestimation was not associated with maternal characteristics including age, education, parity, or SES.[4]

Our study is the first that we are aware of to test a social desirability scale in the context of rural Nepal, though SD scales have been used in urban India[25] and rural Bangladesh.[17] We pretested scale items and made some modifications. The reliability coefficient of our tested scale of 0.58 neared the threshold of 0.60 used to define acceptability, and marginally surpassed that threshold when two items were removed. These values were slightly less than reliability coefficients documented in a study using a lengthier version of the Marlowe-Crowne SDS in four African countries, which varied from 0.63 (Kenya) to 0.80 (Ethiopia).[7] Many of the scale items in our study were positively correlated, however, it is uncertain how well the questions were understood by respondents or the extent to which the scale reflects social desirability in the context of rural Nepal. Additional work to develop and test questions that draw more strongly on local situations and norms would likely further strengthen the ability of the scale to capture social desirability in rural contexts, similar to what has been developed in the Philippines,[29](2) and could enhance the utility of such scales for use in surveys.

A strength of our study is that we used direct observation to assess the receipt of services in clinics. However, the tool used to assess social desirability has not been validated in a rural context, and was only slightly adapted for comprehension by respondents in our area. One adaptation we made to improve comprehension by the study population was to reformat the SDS tool from statements into questions. Ultimately, however, it is not clear how well the scale was able to capture socially desirable response tendencies in this context. Our study used a recall period of approximately 6 months, which may have facilitated more accurate recall of receipt than typically is assessed in surveys such as the DHS, which currently administers ANC modules to women up to 2-years postpartum. The implications of different recall periods for social desirability bias are unclear. Additionally, the enumerators noted the

For peer review only - http://bmjopen.bmj.com/site/about/guidelines.xhtml

presence of others at any point in the interview, and not specifically during the questions about receipt of services; without more detailed information about observer presence when the questions were asked it is difficult to attribute findings specifically to social desirability bias.

These findings reinforce the guidance often given during survey training to conduct surveys without others present, though doing so in practice is often challenging in rural village settings where norms around privacy differ. As we found that the social desirability scale was not well correlated with overestimation or lack of accurate recall, there was no need to correct for social desirability bias. Given that the generalizability of our study to other contexts including other parts of Nepal is uncertain, we recommend further studies be conducted in other contexts to develop and validate social desirability scales and use them to better understand the effects of social desirability bias on estimates of services use.

Acknowledgements

The Nepal Nutrition Intervention Project Sarlahi operates with the permission of and under the auspices of the Social Welfare Council in Nepal and in close conjunction with Nepal Netra Jyoti Singh. This work was funded by the Bill & Melinda Gates Foundation INV007332 and OPP1172551.

Contributorship statement

Conceived study design: MKM, JK, ATL, RAH; Implemented the study: TPL, SL, SKK, EB, PM; Analyzed the data: ATL; Wrote the paper: ATL; Read, provided edits and approved final manuscript: all authors. The authors thank the study team at the Nepal Nutrition Intervention Project-Sarlahi, who conducted household data collection. The Nepal Nutrition Intervention Project Sarlahi operates with the permission

For peer review only

of and under the auspices of the Social Welfare Council in Nepal and in close conjunction with Nepal Netra Jyoti Singh.

Competing interests

The authors declare no competing interests in relation to this work

Funding

This work was funded by the Bill & Melinda Gates Foundation INV007332 and OPP1172551.

Data sharing statement

Date will be made available on reasonable request

Ethics approval statement

This study was approved by the institutional review board at Johns Hopkins Bloomberg School of Public Health (#8808) and by the Nepal Health Research Council (#441 2018). Participants gave informed consent to participate in the study.

References

1 World Health Organization. *WHO recommendations on antenatal care for a positive pregnancy experience*. World Health Organization 2016.

2 Munos MK, Blanc AK, Carter ED, *et al.* Validation studies for population-based intervention coverage indicators: design, analysis, and interpretation. *J Glob Health* 2018;**8**.

3 Hill Z, Okyere E, Wickenden M, *et al.* What can we learn about postnatal care in Ghana if we ask the right questions? A qualitative study. *Glob Health Action* 2015;**8**:28515.

4 Bryce E, Katz J, Munos MK, *et al.* Validation of Maternal Recall of Iron Folic Supplementation During Antenatal Care in Rural Southern Nepal. *Curr Dev Nutr* 2021;**5**:722–722.

5 Bryce E, Katz J, Heidkamp R, *et al.* Validation of maternal report of nutrition-related interventions and counselling during antenatal care in southern Nepal. *Matern Child Nutr* 2021;:e13303.

For peer review only - http://bmjopen.bmj.com/site/about/guidelines.xhtml

6 Stuart GS, Grimes DA. Social desirability bias in family planning studies: a neglected problem. *Contraception* 2009;**80**:108–12.

7 Vu A, Tran N, Pham K, *et al.* Reliability of the Marlowe-Crowne social desirability scale in Ethiopia, Kenya, Mozambique, and Uganda. *BMC Med Res Methodol* 2011;**11**:1–7.

8 Adida CL, Ferree KE, Posner DN, *et al.* Who's asking? Interviewer coethnicity effects in African survey data. *Comp Polit Stud* 2016;**49**:1630–60.

9 Crowne DP, Marlowe D. A new scale of social desirability independent of psychopathology. *J Consult Psychol* 1960;**24**:349.

10 Cagasan Jr L. The development and validation of a Filipino social desirability scale. *Philipp J Psychol* 2016;**49**:19–42.

11 Reynolds WM. Development of reliable and valid short forms of the Marlowe-Crowne Social Desirability Scale. *J Clin Psychol* 1982;**38**:119–25.

12 Paulhus DL. Measurement and control of response bias. 1991.

13 Larson RB. Controlling social desirability bias. *Int J Mark Res* 2019;**61**:534–47.

14 Näher A-F, Krumpal I. Asking sensitive questions: the impact of forgiving wording and question context on social desirability bias. *Qual Quant* 2012;**46**:1601–16.

15 Westerterp KR, Goris AH. Validity of the assessment of dietary intake: problems of misreporting. *Curr Opin Clin Nutr Metab Care* 2002;**5**:489–93.

16 Nguyen PH, Kim SS, Sanghvi T, *et al.* Integrating nutrition interventions into an existing maternal, neonatal, and child health program increased maternal dietary diversity, micronutrient intake, and exclusive breastfeeding practices in Bangladesh: results of a cluster-randomized program evaluation. *J Nutr* 2017;**147**:2326–37.

17 Menon P, Nguyen PH, Saha KK, *et al.* Impacts on breastfeeding practices of at-scale strategies that combine intensive interpersonal counselling, mass media, and community mobilization: results of cluster-randomized program evaluations in Bangladesh and Viet Nam. *PLoS Med* 2016;**13**:e1002159.

18 Ministry of Health, Nepal; New ERA; and ICF. Nepal Demographic and Health Survey 2016. Kathmandu, Nepal: : Ministry of Health, Nepal 2017.

19 Adhikari M, Chalise B, Bista B, *et al.* Sociodemographic correlates of antenatal care visits in Nepal: results from Nepal Demographic and Health Survey 2016. *BMC Pregnancy Childbirth* 2020;**20**:1–10.

20 Mullany LC, Katz J, Li YM, *et al.* Breast-feeding patterns, time to initiation, and mortality risk among newborns in southern Nepal. *J Nutr* 2008;**138**:599–603.

21 World Health Organization. WHO antenatal care randomized trial: manual for the implementation of the new model. World Health Organization 2002.

For peer review only

For peer review only - http://bmjopen.bmj.com/site/about/guidelines.xhtml

22 Spiegelman D and Hertzmark E. Easy SAS calculations for risk or prevalence ratios and differences. American Journal of Epidemiology 2005;**162.3**: 199-200.

23 Liu L, Li M, Yang L, *et al.* Measuring coverage in MNCH: a validation study linking population survey derived coverage to maternal, newborn, and child health care records in rural China. *PLoS One* 2013;**8**:e60762.

24 Benova L, Tunçalp Ö, Moran AC, *et al.* Not just a number: examining coverage and content of antenatal care in low-income and middle-income countries. *BMJ Glob Health* 2018;**3**:e000779.

25 Fisher RJ, Katz JE. Social-desirability bias and the validity of self-reported values. *Psychol Mark* 2000;**17**:105–20.

26 Crowne DP, Crowne DP, Marlowe D. *The approval motive: Studies in evaluative dependence*. New York: Wiley 1964.

27 Thorne-Lyman AL, Lama TP, Heidkamp RA, *et al.* Cognitive testing of questions about antenatal care and nutrition interventions in southern Nepal. *Soc Sci Med* In Press.

28 World Health Organization. Developing and validating an iron and folic acid supplementation indicator for tracking progress towards global nutrition monitoring framework targets: final report June 2018. 2018.

29 Boerma T, Requejo J, Victora CG, *et al.* Countdown to 2030: tracking progress towards universal coverage for reproductive, maternal, newborn, and child health. *The Lancet* 2018;**391**:1538–48.

30 Ranjan A, George TS. Evaluating social desirability bias in assessment. *Indian J Health Wellbeing* 2014;**5**:948.

For peer review only

For peer review only - http://bmjopen.bmj.com/site/about/guidelines.xhtml

Supplementary Materials

Supplementary Figure 1. Study flow chart

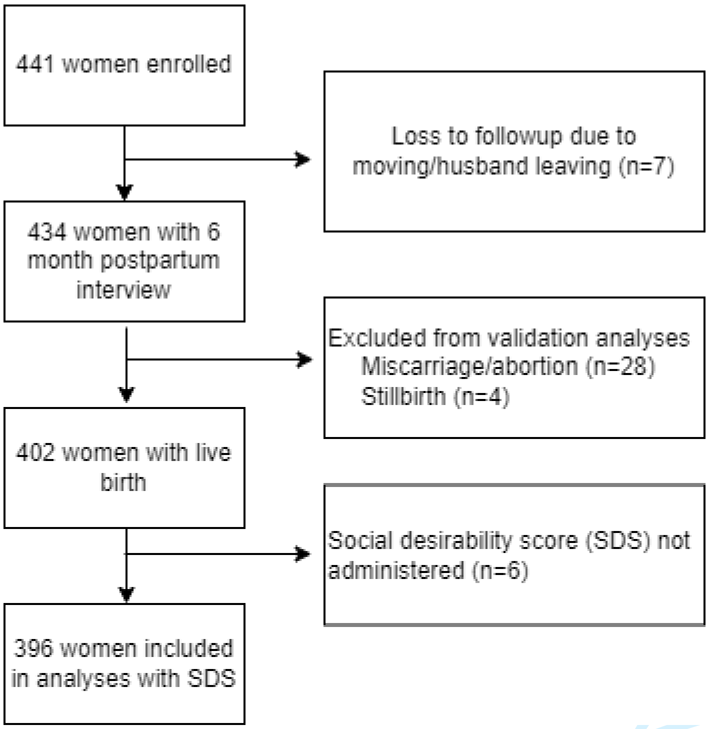

Supplementary Tables

Supplementary Table 1. Questions included in the survey and their origins[1]

Question	Responses	Origin/notes
21. How many times did you receive antenatal care during this pregnancy?	Number of times:<br>01-15 times (go to 21.a.)<br>99= Don't know (go to 22)	2016 Demographic and Health Survey, DHS-8
23. During this pregnancy, were you given or did you buy any iron/folic acid tablets?<br><br>Show photo of tablets	0= No (go to 24)<br>1= Yes (go to 23.a.)<br>9= Don't Know (go to 24)	2016 Demographic and Health Survey, DHS-8
23.a. During the whole pregnancy, for how many days did you take the tablets?<br>If answer is not numeric, probe for approximate number of days	Days 000-270<br>999=Don't know	2016 Demographic and Health Survey, DHS-8
27. During this pregnancy, at any antenatal visit, did you receive any information about nutrition or diet?	0= No (go to 28)<br>1= Yes (go to 27.a.)<br>9= Don't Know (go to 28)	Original question
27.b. What information or messages did you receive during your pregnancy about nutrition or diet?<br><br>First allow respondent to provide any answers and check all that apply<br><br>Then probe to identify each response and record all mentioned<br>[ask each answer individually]	Were you told to…<br><br>□ Eat more (quantity)<br>□ Eat a variety of foods rich with iron, vitamin A or C<br>□ Take iron tablets (IFA's)<br>□ Take calcium tablets<br>□ How to manage nausea/vomiting<br>□ Other (specify) ______<br><br>0= No<br>1= Yes, without prompting<br>2= Yes, with prompting<br>9= Don't know	Original question

[1]These questions were administered to respondents approximately 6 months after delivery, asking them to recall services received during the most recent pregnancy. The responses column lists the possible response categories for each question and the origin column describes the source of the question.

For peer review only

Supplementary Table 2. List of questions included from the short form Marlowe Crown social desirability index, inclusion in the score

Code	Question	% Yes	Negative key[1]
SM1	Do you sometimes find it hard to go on with your work if you are not encouraged?	39.6	No
SM2	Do you sometimes feel resentful when you don't get your way?	10.0	No
SM3	Do you occasionally give up doing something because you don't think you have the ability	31.1	No
SM4	Are there any times when you felt like rebelling against people in authority even though you knew they were right?	59.9	No
SM5	Are you always a good listener no matter who you are talking to?	21.9	Yes
SM6	Are there any occasions when you took advantage of someone?	89.1	No
SM7	When you make a mistake are you always willing to admit it?	64.7	Yes
SM8	Are you always courteous, even to people who are disagreeable/not pleasant?	19.5	Yes
SM9	Have you ever been irked when people expressed ideas very different from your own?	14.4	No
SM10	Are there any times when you were quite jealous of the good fortune of others?	85.1	No
SM11	Do you sometimes get irritated/annoyed by people who ask you to do something for them?	27.6	No
SM12	Have you ever deliberately said something that hurt someone's feelings	89.5	No

For peer review only

Supplementary Table 3. Correlations between questions used for assessment of the social desirability

		SM1	SM2	SM3	SM4	SM5	SM6	SM7	SM8	SM9	SM10	SM11	SM12
SM1	Do you sometimes find it hard to go on with your work if you are not encouraged?	1.00											
SM2	Do you sometimes feel resentful when you don't get your way?	0.31 ***	1.00										
SM3	Do you occasionally give up doing something because you don't think you have the ability	0.07	0.08	1.00									
SM4	Are there any times when you felt like rebelling against people in authority even though you knew they were right?	-0.03	0.10 *	-0.08	1.00								
SM5	Are you always a good listener no matter who you are talking to?	0.15 **	0.16 **	0.03	0.22 ***	1.00							
SM6	Are there any occasions when you took advantage of someone?	.02	.01	-0.01	0.11 *	-0.03	1.00						
SM7	When you make a mistake are you always willing to admit it?	.08	0.04	-0.12 *	0.36 ***	0.23 ***	-0.04	1.00					
SM8	Are you always courteous, even to people who are disagreeable/not pleasant?	.21 ***	0.21 ***	0.03	0.03	0.38 ***	-0.03	0.21 ***	1.00				
SM9	Have you ever been irked when people expressed ideas very different from your own?	.20 ***	0.34 ***	-0.03	0.15 **	0.16 **	0.07	0.13 *	0.32 ***	1.00			
SM10	Are there any times when you were quite jealous of the good fortune of others?	0.16 **	0.07	0.04	0.15 *	0.07	0.06	0.15 **	0.05	0.11 *	1.00		
SM11	Do you sometimes get irritated/annoyed by people who ask you to do something for them?	.29 ***	0.34 ***	0.01	0.15 **	0.28 ***	-0.02	0.21 ***	0.32 ***	0.29 ***	0.15 **	1.00	
SM12	Have you ever deliberately said something that hurt someone's feelings	-0.11 *	-0.02	0.005	0.02	0.07	0.17 **	-0.04	-0.02	-0.07	0.06	0.03	1.00

***p<0.0001, **p<0.01 *p<0.05

For peer review only

Supplementary Table 4. Characteristics of women and households by presence of others during the interview

	No other adults present (n=195)	Any other adult present (n=207)	Husband present (n=28)	Mother in law present (n=98)	Friend present (n=50)	Overall (n=402)
Women's age, years (mean, sd)	22.7 (4.3)	22.3 (4.1)	20.6 (2.9)	21.8 (3.7)	23.4 (4.8)	22.5 (4.2)
# Observed ANC visits	5.0 (2.6)	4.3 (2.3)	4. 1 (2.1)	4.4 (2.5)	4.4 (2.3)	4.7 (2.5)
Socioeconomic status quartile %						
1 (Bottom)	34.9	38.7	32.1	36.7	40.0	36.8
2	18.0	17.4	21.4	17.4	16.0	17.7
3	31.3	31.4	32.1	29.6	38.0	31.3
4 (Top)	15.9	12.6	14.3	16.3	6.0	14.2
Any prior live births (%)	70.3	67.2	60.7	59.2	74.0	68.7
Any formal education (%)	42.1	38.6	42.9	44.9	24.0	40.3
Trimester at enrollment						
1-3 months	46.2	38.7	28.6	40.8	34.0	42.3
4-6 months	51.8	59.9	71.4	57.1	66.0	56.0
7-9 months	2.1	1.5	0	2.0	0	1.7
Got help during interview*	0	52.0	50.0	48.1	32.7	19.9

For peer review only

Supplementary Table 5. Receipt of help during the interview and accuracy of recall of services

	Received help during interview from another adult ARR (95% CI)	Received help from MIL during the interview ARR (95% CI)
Deworming	0.97 (0.78, 1.20)	0.94 (0.78, 1.13)
Receipt of Ca or Ca info	0.99 (0.82, 1.20)	1.01 (0.90, 1.13)
Counseling on		
Nutrition	0.97 (0.85, 1.11)	No convergence
Eating more	0.93 (0.77, 1.12)	No convergence
Diverse diet	No convergence	No convergence
Managing nausea	1.02 (0.70, 1.49)	1.18 (0.91, 1.53)
Not drinking *paan* alcohol	0.94 (0.64, 1.38)	1.24 (1.00, 1.55)
Not smoking/ using	0.88 (0.60, 1.29)	1.14 (0.92, 1.41)
Accuracy of IFA estimate		
Within 30 tablets	1.08 (0.69, 1.69)	1.16 (0.81, 1.64)
Within 60 tablets	1.04 (0.80, 1.36)	1.07 (0.85, 1.34)
Within 90 tablets	1.05 (0.89, 1.24)	1.09 (0.96, 1.25)
Estimate number of ANC visits		
Exactly	0.47 (0.16, 1.38)	0.76 (0.39, 1.49)
Within 1 visit	0.86 (0.57, 1.29)	0.81 (0.58, 1.13)
Within 2 visits	0.98 (0.78, 1.23)	1.02 (0.86, 1.21)

For peer review only

STROBE Statement—checklist of items that should be included in reports of observational studies

	Item No	Recommendation	Page No
**Title and abstract**	1	(*a*) Indicate the study's design with a commonly used term in the title or the abstract	2
		(*b*) Provide in the abstract an informative and balanced summary of what was done and what was found	2
**Introduction**			
Background/rationale	2	Explain the scientific background and rationale for the investigation being reported	4
Objectives	3	State specific objectives, including any prespecified hypotheses	5
**Methods**			
Study design	4	Present key elements of study design early in the paper	6-7
Setting	5	Describe the setting, locations, and relevant dates, including periods of recruitment, exposure, follow-up, and data collection	6
Participants	6	(*a*) *Cohort study*—Give the eligibility criteria, and the sources and methods of selection of participants. Describe methods of follow-up<br>*Case-control study*—Give the eligibility criteria, and the sources and methods of case ascertainment and control selection. Give the rationale for the choice of cases and controls<br>*Cross-sectional study*—Give the eligibility criteria, and the sources and methods of selection of participants	8
		(*b*) *Cohort study*—For matched studies, give matching criteria and number of exposed and unexposed<br>*Case-control study*—For matched studies, give matching criteria and the number of controls per case	
Variables	7	Clearly define all outcomes, exposures, predictors, potential confounders, and effect modifiers. Give diagnostic criteria, if applicable	6-9
Data sources/ measurement	8*	For each variable of interest, give sources of data and details of methods of assessment (measurement). Describe comparability of assessment methods if there is more than one group	6-8
Bias	9	Describe any efforts to address potential sources of bias	Entire paper
Study size	10	Explain how the study size was arrived at	7
Quantitative variables	11	Explain how quantitative variables were handled in the analyses. If applicable, describe which groupings were chosen and why	9
Statistical methods	12	(*a*) Describe all statistical methods, including those used to control for confounding	9
		(*b*) Describe any methods used to examine subgroups and interactions	na
		(*c*) Explain how missing data were addressed	9
		(*d*) *Cohort study*—If applicable, explain how loss to follow-up was addressed<br>*Case-control study*—If applicable, explain how matching of cases and controls was addressed<br>*Cross-sectional study*—If applicable, describe analytical methods taking account of sampling strategy	9, Supp fig 1

For peer review only - http://bmjopen.bmj.com/site/about/guidelines.xhtml

(*e*) Describe any sensitivity analyses

Continued on next page

For peer review only

For peer review only - http://bmjopen.bmj.com/site/about/guidelines.xhtml

**Results**			
Participants	13*	(a) Report numbers of individuals at each stage of study—eg numbers potentially eligible, examined for eligibility, confirmed eligible, included in the study, completing follow-up, and analysed	Sup Fig 1, 10
		(b) Give reasons for non-participation at each stage	Supp fig 1
		(c) Consider use of a flow diagram	Supp fig 1
Descriptive data	14*	(a) Give characteristics of study participants (eg demographic, clinical, social) and information on exposures and potential confounders	Table 1; supp table 4
		(b) Indicate number of participants with missing data for each variable of interest	tables
		(c) *Cohort study*—Summarise follow-up time (eg, average and total amount)	Page 8
Outcome data	15*	*Cohort study*—Report numbers of outcome events or summary measures over time	*tables*
		Case-control study—Report numbers in each exposure category, or summary measures of exposure	
		Cross-sectional study—Report numbers of outcome events or summary measures	
Main results	16	(*a*) Give unadjusted estimates and, if applicable, confounder-adjusted estimates and their precision (eg, 95% confidence interval). Make clear which confounders were adjusted for and why they were included	No room for unadjusted.
		(*b*) Report category boundaries when continuous variables were categorized	9
		(*c*) If relevant, consider translating estimates of relative risk into absolute risk for a meaningful time period	na
Other analyses	17	Report other analyses done—eg analyses of subgroups and interactions, and sensitivity analyses	Supp tables
**Discussion**			
Key results	18	Summarise key results with reference to study objectives	10-11
Limitations	19	Discuss limitations of the study, taking into account sources of potential bias or imprecision. Discuss both direction and magnitude of any potential bias	13-14
Interpretation	20	Give a cautious overall interpretation of results considering objectives, limitations, multiplicity of analyses, results from similar studies, and other relevant evidence	13-14,
Generalisability	21	Discuss the generalisability (external validity) of the study results	14
**Other information**			
Funding	22	Give the source of funding and the role of the funders for the present study and, if applicable, for the original study on which the present article is based	1

*Give information separately for cases and controls in case-control studies and, if applicable, for exposed and unexposed groups in cohort and cross-sectional studies.

Note: An Explanation and Elaboration article discusses each checklist item and gives methodological background and published examples of transparent reporting. The STROBE checklist is best used in conjunction with this article (freely available on the Web sites of PLoS Medicine at http://www.plosmedicine.org/, Annals of Internal Medicine at http://www.annals.org/, and Epidemiology at http://www.epidem.com/). Information on the STROBE Initiative is available at www.strobe-statement.org.

For peer review only

BMJ Open

How does social desirability bias influence survey-based estimates of the use of antenatal care in rural Nepal? A validation study

Journal:	*BMJ Open*
Manuscript ID	bmjopen-2022-071511.R2
Article Type:	Original research
Date Submitted by the Author:	05-Jul-2023
Complete List of Authors:	Thorne-Lyman, Andrew; Johns Hopkins University Bloomberg School of Public Health, International Health<br>Lama, Tsering; Johns Hopkins University Bloomberg School of Public Health, International Health; Nepal Nutrition Intervention Project, Sarlahi<br>Heidkamp, Rebecca; Johns Hopkins University Bloomberg School of Public Health, International Health<br>Munos, Melinda; Johns Hopkins University, Bloomberg School of Public Health, Department of International Health<br>Manandhar, Porcia; Johns Hopkins University Bloomberg School of Public Health, International Health<br>Khatry, Subarna; Nepal Nutr Intervent Project Sarlahi; Johns Hopkins University Bloomberg School of Public Health, International Health<br>Bryce, Emily; Johns Hopkins University Bloomberg School of Public Health, International Health<br>LeClerq, Steven C.; Johns Hopkins Bloomberg School of Public Health, Department of International Health; Nepal Nutrition Intervention Project<br>Katz, Joanne; Johns Hopkins University Bloomberg School of Public Health, International Health
**Primary Subject Heading**:	Global health
Secondary Subject Heading:	Health services research, Nutrition and metabolism, Public health, Research methods
Keywords:	Nutrition < TROPICAL MEDICINE, PRIMARY CARE, PUBLIC HEALTH, OBSTETRICS

SCHOLARONE™
Manuscripts

BMJ

I, the Submitting Author has the right to grant and does grant on behalf of all authors of the Work (as defined in the below author licence), an exclusive licence and/or a non-exclusive licence for contributions from authors who are: i) UK Crown employees; ii) where BMJ has agreed a CC-BY licence shall apply, and/or iii) in accordance with the terms applicable for US Federal Government officers or employees acting as part of their official duties; on a worldwide, perpetual, irrevocable, royalty-free basis to BMJ Publishing Group Ltd ("BMJ") its licensees and where the relevant Journal is co-owned by BMJ to the co-owners of the Journal, to publish the Work in this journal and any other BMJ products and to exploit all rights, as set out in our licence.

The Submitting Author accepts and understands that any supply made under these terms is made by BMJ to the Submitting Author unless you are acting as an employee on behalf of your employer or a postgraduate student of an affiliated institution which is paying any applicable article publishing charge ("APC") for Open Access articles. Where the Submitting Author wishes to make the Work available on an Open Access basis (and intends to pay the relevant APC), the terms of reuse of such Open Access shall be governed by a Creative Commons licence – details of these licences and which Creative Commons licence will apply to this Work are set out in our licence referred to above.

Other than as permitted in any relevant BMJ Author's Self Archiving Policies, I confirm this Work has not been accepted for publication elsewhere, is not being considered for publication elsewhere and does not duplicate material already published. I confirm all authors consent to publication of this Work and authorise the granting of this licence.

For peer review only

Title: How does social desirability bias influence survey-based estimates of the use of antenatal care in rural Nepal? A validation study

Authors: Andrew L. Thorne-Lyman[a], Tsering P. Lama[a,b], Rebecca A. Heidkamp[a], Melinda K. Munos[a], Porcia Manandhar[a], Subarna K. Khatry[a,b], Emily Bryce[a] Steven C. LeClerq[a,b], Joanne Katz[a]

[a]Department of International Health, Johns Hopkins Bloomberg School of Public Health, Baltimore, MD, 21205, USA

[b]Nepal Nutrition Intervention Project-Sarlahi, Kathmandu, Nepal

Corresponding author: Andrew L. Thorne-Lyman, Program in Human Nutrition, Department of International Health, 615 N. Wolfe Street E2545, Johns Hopkins Bloomberg School of Public Health, Baltimore, Maryland, USA. **Email:** athorne1@jhu.edu, **Phone**: +1 856 214-5755, **Fax**:+1 410-955-0196

Keywords: Nepal, survey, nutrition, social desirability bias, antenatal care, recall, validity

For peer review only

For peer review only - http://bmjopen.bmj.com/site/about/guidelines.xhtml

Abstract

Objectives: Social desirability bias is often speculated to influence survey responses but seldom studied in health care. The objective was to explore whether social desirability scores (SDS) or the presence of interview observers is associated with inaccurate recall and overestimation of antenatal care (ANC) services.

Design: Longitudinal validation study comparing recalled receipt of ANC services and nutrition components of ANC against direct observations of care. An adapted short form Marlowe-Crowne questionnaire was used to generate a social desirability score and the presence of interview observers was treated as a separate exposure. We assessed accuracy and overestimation of recalled receipt of ANC services against observed receipt using log-binomial regression, adjusting for age, education, first-pregnancy, and socio-economic status.

Setting: Rural Southern Nepal with recruitment from five government health posts

Participants: 401 pregnant women

Results: Social desirability scores did not significantly predict accuracy or overestimation of most types of ANC care except counselling on nausea. Higher SDS was associated with more accurate recall [adjusted RR, aRR 1.08 (95% CI 1.03, 1.12)] and less overestimation [aRR 0.85 (0.80, 0.91)]. The presence of mothers in law or husbands during interviews was associated with greater overestimation of the number of ANC visits received by more than 3 visits [aRR 2.07 (1.11, 3.84)] and [aRR 4.19 (2.17, 8.10)] respectively. Those interviewed with friends present tended to overestimate the receipt of counselling on nausea, avoiding alcohol, and not smoking.

Conclusion: The presence of observers can lead to overestimation of the receipt of ANC care and support the conduct of interviews in private settings despite challenges of doing so in village settings.

For peer review only - http://bmjopen.bmj.com/site/about/guidelines.xhtml

Findings that the SDS did not predict the accuracy of most types of ANC care might reflect a reality that such questions may not be sensitive from a social-norms perspective. Additional local adaptation of SDS is recommended.

Strengths and limitations of the study

- The effect of social desirability bias and presence of interview observers on response accuracy was evaluated against the gold standard of observed receipt of antenatal care services.
- The tool used to assess social desirability was adapted for comprehension for the local population in rural Nepal, but has not been validated for that context.
- Interviewers noted the presence of others at any point during the interview, and not specifically during specific portions of the interview, likely attenuating estimates of the relationship between presence of others and validity of reported services receipt.

For peer review only

For peer review only - http://bmjopen.bmj.com/site/about/guidelines.xhtml

Introduction

The World Health Organization (WHO) recommends a number of nutrition interventions in pregnancy, including iron-folic acid supplements (IFA), counselling on healthy eating and physical activity, and in certain contexts calcium, micronutrient supplements and balanced energy and protein supplements.[1] Large-scale household surveys, including those implemented by the Demographic Health Survey program, are used across low and middle-income countries to measure coverage of antenatal interventions in order to monitor progress towards targets and evaluate policies and programs.[2]

Many survey-based coverage indicators come from self-reported data and a growing number of studies have explored the validity of responses to questions about the receipt of nutrition interventions delivered through antenatal care.[2][3] A study carried out by our research team in Nepal found that respondents tend to overestimate the receipt of IFA in pregnancy, and that overestimation was not explained by either maternal or household characteristics.[4] Women's reports of the receipt of various counselling interventions, weight measurement, and deworming were generally recalled more accurately than questions about other services such as counselling to not drink alcohol or tobacco.[5]

Social desirability response bias (SDB) is a common type of bias that reflects the tendency of respondents to reply in a manner that may be viewed positively by social peers or that is consistent with social norms and expectations. Social desirability bias (SDB) is generally thought to be greater for questions that are perceived as sensitive or subject to judgment such as those related to sexual practices or family planning.[6,7] The perceived relationships between respondents and interviewers or observers can also influence socially desirable responses. For example, a study administered in 14 African countries found that respondents give systematically different answers to interviewers depending on whether or not they were from their same ethnic group.[8]

For peer review only - http://bmjopen.bmj.com/site/about/guidelines.xhtml

Survey-based scales to measure social desirability were developed in the field of social psychology in the 1960's beginning with the 33-question Marlowe-Crowne scale[9]. Shorter versions of these scales have been shown to be reliable across different cultural contexts, though more work is needed to refine and test them.[7,10, 11]. These scales include a variety of positively keyed questions that are "culturally acceptable but improbable" or "desirable but rare" and negatively keyed items that are "culturally unacceptable but probable" or "undesirable but common".[10] The scales are used to detect and quantify the extent of socially desirable responses in surveys. Where it has been detected, a number of approaches exist correct for it or mitigate its effects including a posteriori statistical adjustment for SDS,[12,13] excluding high SDS scorers[13] or rephrasing questions using forgiving wording[14].

Social desirability bias has been frequently described as an area of potential concern in nutrition-related studies collecting data about diet, physical activity, and self-reported weight, but few studies have directly quantified the strength of association between social desirability scores and these domains.[15] Evaluations of the Alive & Thrive infant and young child feeding program in Vietnam and Bangladesh explored whether adjusting for short-form social desirability scales affected estimates of program impact on diet, but found that results were unchanged.[16,17] To our knowledge, no previous studies have directly explored whether SDB or presence of others during survey interviews is a potential explanation for inaccurate survey responses about the receipt of ANC and related nutrition interventions. The aims of this study were therefore to:

1. Adapt and test a modified short-form version of the Marlow-Crowne social desirability scale in rural Nepal.
2. Explore whether social desirability or the presence of other adults during interviews helps to explain previous findings related to overestimation of the receipt of services.

For peer review only - http://bmjopen.bmj.com/site/about/guidelines.xhtml

3. Explore whether social desirability or the presence of other adults during interviews is associated with inaccurate recall of ANC and associated services.

Methods

Study area and population

This study took place in Sarlahi District, an area in the southern plains (*Tarai)* of Nepal located in Province 2, the province with the lowest rates of ANC coverage in Nepal. According to the 2016 Demographic and Health Survey, only 36% of women in Province 2 received all four recommended ANC visits, far below the national average of 59%.[18]. These low rates appear paradoxical given that 70% of the population in Sarlahi lived within 30 minutes of a government health facility. This paradox may be explained in part by low rates of maternal education and gender norms that limit women's ability to travel outside of the household.[19] The population of Sarlahi is linguistically and culturally heterogeneous, including both Nepali and Maithili-speaking groups.[20]

Antenatal care and nutrition services delivered through ANC

At the time of our study, Nepal was implementing the World Health Organization's focused ANC (FANC) model, with recommended visits occurring in the 4th, 6th, 8^{th,} and 9th months of pregnancy. The government incentivizes ANC participation by providing 800 rupees (~7 USD) to women who complete all four visits.[21] Nutrition interventions delivered through ANC in Nepal include the provision of iron-folic acid supplements, checking of weight, and counselling on various topics related to nutrition and breastfeeding.

Study design

This study was part of a larger effort to validate coverage indicators for various nutrition services through ANC, described in detail elsewhere.[4,5] In brief, study staff were based at five government

For peer review only - http://bmjopen.bmj.com/site/about/guidelines.xhtml

health posts and trained to directly observe and record the receipt of ANC services by enrolled women. The observers' 28-item checklist included details about the provision of iron-folic acid pills (e.g. how many pills women received, whether they were instructed to purchase iron folic acid tablets or syrup), measurement of weight, and types of counselling on nutrition and breastfeeding. These direct observations served as a "gold standard" to validate maternal recall of ANC services received at study clinics. Maternal recall was assessed by interviewing enrolled women using a structured questionnaire around six months after delivery. [2,4,5] The interview questions were drawn from the official translated Nepali DHS 2016 questionnaire (DHS Round 7), a draft of the DHS Round 8 questionnaire, and novel questions to assess different components of counselling and weight gain assessment (**Supplementary Table 1)**. The six-month visits were conducted at the women's homes or their parental homes. Women were asked about socioeconomic status at enrollment to the validation study and during the visit at six months after delivery. As described in detail elsewhere [4], a target sample size of 300 women was established for the study based on an assumption of 50% coverage for receipt of IFA and lower coverage for counselling. After accounting for loss-to-follow-up, the possibility of women going elsewhere for ANC, and of non-live birth outcomes, a target study enrollment of 450 women was established.

Measurement of social desirability

Questions from a short-form Marlowe-Crowne social desirability index (**Supplementary Table 2)** were translated and back-translated by Nepali/English speakers familiar with the study context, pre-tested and slightly adapted to the local context. During pre-testing one of the questions was not well understood and dropped; 12 questions were included in the six-month visit questionnaire.[11] Study staff also recorded whether any other adult had been present at any point during the interview (with specific codes for husbands, mothers-in law, friends of the respondent or other adults), as well as whether those present had helped the respondent to answer any questions.

For peer review only - http://bmjopen.bmj.com/site/about/guidelines.xhtml

Correlations between different items of the social desirability index were explored after reverse coding negatively keyed questions, and most were found to be positively associated with one another (**Supplementary Table 3**). Tests of internal consistency revealed the full 12-item scale to have a Cronbach's alpha of 0.58 and only minor improvements (to an alpha of 0.65) were possible by removing several items so the full 12-item scale was used in analyses. On visual inspection, scores on the social desirability scale (SDS) were only slightly skewed, with a mean of 5.5 and a standard deviation of 2.1.

Data collection

Enrollment criteria for the parent trial included currently pregnant, married women aged 15 years or older, who a) resided in the study area, b) visited one of the five health posts for their first ANC visit and c) had the intention to return to these study sites for subsequent ANC visits. All interview questions were translated and back-translated by Nepali/English speakers familiar with the study context. These questions were pre-tested and minor changes were made to help facilitate comprehension in the local context. Both Maithili and Nepali-speaking interviewers conducted the interviews following training exercises designed to standardize the approach to translation. Interviewers were trained to replicate usual survey conditions. This included asking respondents to answer questions themselves even if a family member had tried to answer it for her and repeating or rephrasing questions in a simpler way if respondents were confused or responded with "I don't know".

The study started in December 2018 and reached full enrollment by November 2019. Direct observations of ANC visits were nearly completed by March 2020 when a shut-down for the Covid-19 pandemic interrupted all non-emergency health services in Nepal. Data collection for the post-partum six-month visit was started in September 2019 but Covid-19 related shutdowns caused delays. The mean number of days that passed from delivery to the post-partum data collection visit was 252 with a standard deviation of 75 days. Precautions taken to prevent the spread of Covid-19 included masking by

For peer review only - http://bmjopen.bmj.com/site/about/guidelines.xhtml

interviewers and respondents, handwashing with soap provided to interviewees, holding interviews outdoors when weather permitted, and maintaining a distance of more than six feet during data collection.

Data analysis

We examined whether SDS or presence of other adults during interviews was associated with the accuracy or overestimation of recalled receipt of specific ANC services using log-binomial bivariate and multivariable regressions to directly estimate risk ratios or log-Poisson models in the case of model non-convergence [22]. Multivariable analyses adjusted for age (continuous), education (any vs. none), first pregnancy (yes/no), and quartile of socioeconomic status as possible confounders. We used a theory-based approach to selection of covariates for these models. For interpretation purposes, high SDS corresponds to women that would be more likely to be exhibit socially desirable responses. SDS was treated as both a continuous score and as a dichotomous variable in analyses, with the upper quartile defined as those with a score of 7 or greater compared against the lower three quartiles, recognizing that socially desirable response bias may not be a linear construct. For receipt of counselling interventions, calcium, and deworming, accurate recall was defined as alignment (yes/no) of the follow-up interview responses with observed receipt or non-receipt of those interventions.[4,5] Overestimation for these interventions was defined as respondents that reported receipt but were not observed receiving it, and for each intervention women were categorized as over- estimators or non-over-estimators. For ANC visits and IFA, several different thresholds were used to define accuracy and the degree of overestimation. For reasons of statistical power, we restricted our analysis to questions about interventions with directly observed coverage below 90%. A complete case analysis was used for the treatment of missing data and loss to follow-up was minimal (**Supplementary Figure 1**).

IRB approval

For peer review only - http://bmjopen.bmj.com/site/about/guidelines.xhtml

This research conformed with the principles embodied in the Declaration of Helsinki. The institutional review boards at Johns Hopkins Bloomberg School of Public Health and the Nepal Health Research Council both approved this study including permission to collect and resume data collection during the Covid-19 epidemic. Signed consent was obtained from all participants. Patients and the public were not involved in the design, conduct, reporting or dissemination plans of this research. Dissemination and discussion of study design and results is made to a national committee and a local district committee.

Patient and public involvement

The research questions were not informed through direct consultation of patients' priorities, experience or preferences. However, many members of our local research team are from the local population where the research is conducted and were involved in pretesting, questionnaire development and recruitment. Findings will be disseminated to national and local stakeholders through meetings and local presentations.

Results

Of the 441 women enrolled in the study, 396 were included in our analysis after accounting for exclusion criteria described in **Supplementary Figure 1**. The mean age, number of observed ANC visits, and birth history were similar among women in the upper quartile of SDS compared those in the lower three quartiles (**Table 1)**. Women with lower SDS tended to have lower education and enrolled in the study later in pregnancy than those with higher scores. Twice as many women with lower SDS had another adult present during the interview than those with higher scores; mothers-in-law and other adults were the most common observers. Those with lower SDS were only slightly more likely to have an observer help them respond to interview questions. Characteristics of women disaggregated by the presence of observers are presented in **Supplementary Table 4**; those with a friend present were less likely to be in the top quartile of socioeconomic status or to have education.

For peer review only - http://bmjopen.bmj.com/site/about/guidelines.xhtml

210 Table 1. Characteristics of the study population

	Bottom 3 quartiles SDS (n=284)	Upper quartile SDS (n=112)	Overall (n=396)
Age, years, mean (sd)	22.7 (4.3)	21.9 (4.0)	22.5 (4.2)
# Observed ANC visits, mean (sd)	4.6 (2.4)	4.8 (2.6)	4.7 (2.5)
Socioeconomic status quartile (%)			
1 (Bottom)	36.6	39.3	37.4
2	17.6	17.0	17.4
3	31.7	29.5	31.1
4 (Top)	14.1	14.3	14.1
Any prior live births (%)	71.7	71.8	71.7
Any formal education (%)	32.2	22.3	28.3
Trimester at enrollment (%)			
1-3 months	39.4	50.0	42.4
4-6 months	59.2	47.3	55.8
7-9 months	1.4	2.7	1.8
Interview observed by another adult (%)	60.9	31.1	52.1
Husband	7.4	5.9	7.0
Mother-in-law	28.3	14.3	24.4
Friend	15.9	5.0	12.9
Other adult	39.5	16.0	32.9
Observer helped respondent answer questions (%)	29.0	23.5	27.9

211

212 About half of women overestimated the number of IFA tablets they received by at least 30 tablets and
213 over a third overestimated the number of ANC visits by at least one visit **(Table 2**). About a third of
214 women reported receiving counselling for nausea, alcohol or smoking that had not been observed at
215 clinics. Null relations were apparent between SDS and overestimation of receipt of most services,
216 although some associations neared statistical significance when SDS was treated as a dichotomous
217 predictor. Additionally, the dichotomous SDS had a stronger relation with extreme overestimation of
218 reported receipt of both ANC and IFA compared to more modest overestimation. SDS were generally
219 not associated with accuracy of recalled services (**Table 3**). The only exception was for counselling on
220 the management of nausea: women with higher SDS tended to have more accurate recall.

221

For peer review only - http://bmjopen.bmj.com/site/about/guidelines.xhtml

For peer review only

Table 2. High social desirability score as a predictor of overestimation of nutrition/ANC services receipt in pregnancy

	N	Proportion Overestimating %	SDS[2] as a predictor of overestimation	
			Dichotomous SDS adjusted[1,3] RR (95% CI)	Continuous SDS adjusted RR[1,3] (95% CI)
Deworming	395	15.2	1.12 (0.67, 1.87)	1.01 (0.90, 1.13)
Receipt or prescribe Ca	390	7.6	0.76 (0.33, 1.75)	0.90 (0.75, 1.07)
Counselling on				
Managing nausea	391	38.3	0.49 (0.34, 0.72)	0.85 (0.80, 0.91)
Not drinking alcohol	395	37.7	1.26 (0.97, 1.63)	1.04 (0.98, 1.10)
Not smoking/ using *paan*	395	31.2	1.23 (0.91, 1.66)	1.02 (0.95, 1.09)
Overestimate IFA receipt				
By >30 tablets	404	51.7	1.07 (0.87, 1.30)	1.02 (0.97, 1.06)
By >60 tablets	404	34.0	1.16 (0.87, 1.54)	1.03 (0.97, 1.10)
By >90 tablets	404	17.5	1.28 (0.83, 1.98)	1.02 (0.93, 1.13)
Overestimate # of ANC visits				
By >1 visit	402	34.8	0.99 (0.74, 1.34)	1.00 (0.94, 1.07)
By >2 visits	402	18.7	1.17 (0.75, 1.82)	1.02 (0.92, 1.13)
By >3 visits	402	9.2	1.37 (0.71, 2.62)	1.02 (0.87, 1.18)

[1]Adjusted risk ratio > 1 indicates greater risk of overestimating receipt of services

[2]SDS=Social desirability score, treated as upper quartile vs lower three quartiles

[3]Adjusted for woman's age (continuous), any education, first pregnancy, and socio-economic status quartile

Table 3. High social desirability score as a predictor of accurate recall of nutrition/ANC services receipt in pregnancy

	N	Proportion accurate %	SDS[2] as a predictor of accurate recall	
			Dichotomous SDS adjusted[1,3] RR (95% CI)	Continuous SDS adjusted RR[1,3] (95% CI)
Deworming	395	77.5	0.97 (0.86, 1.09)	0.99 (0.96, 1.01)
Receipt or prescribe Ca	390	85.6	0.98 (0.89, 1.09)	0.99 (0.97, 1.01)
Counselling on				
Managing nausea	391	50.9	1.30 (1.07, 1.60)	1.08 (1.03, 1.12)
Not drinking alcohol	395	55.7	0.89 (0.72, 1.10)	0.97 (0.93, 1.02)
Not smoking/ using *paan*	395	59.5	0.91 (0.74, 1.11)	0.98 (0.94, 1.02)
Overestimate IFA receipt				
By >30 tablets	396	39.9	0.87 (0.66, 1.16)	0.98 (0.93, 1.04)
By >60 tablets	396	61.6	1.01 (0.85, 1.20)	1.02 (0.98, 1.05)
By >90 tablets	396	79.3	0.91 (0.80, 1.03)	1.00 (0.97, 1.02)
Overestimate # of ANC visits				
By >1 visit	396	21.0	0.94 (0.60, 1.45)	1.01 (0.92, 1.11)
By >2 visits	396	55.1	0.91 (0.74, 1.12)	1.02 (0.97, 1.06)
By >3 visits	396	75.5	0.96 (0.84, 1.10)	1.01 (0.98, 1.04)

For peer review only

For peer review only - http://bmjopen.bmj.com/site/about/guidelines.xhtml

[1]Adjusted risk ratio > 1 indicates more accurate recall

[2]SDS=Social desirability score, treated as upper quartile vs lower three quartiles

[3]Adjusted for woman's age (continuous), any education, first pregnancy, and socio-economic status quartile

The presence of any other adult was significantly associated with overestimation of receipt of counselling on managing nausea as well as overestimation of the number of ANC visits. Mothers-in-law (MILs) were present during about a quarter of all interviews. Their presence was associated with significant overestimation of the receipt of deworming tablets [ARR 1.90 (95% C.I. 1.18, 3.05)] and more than doubled the risk of overestimating the number of ANC visits by 3 or more visits [ARR 2.07 (95% 1.11, 3.84)] **(Table 4)**. Interestingly, the presence of a MIL during the interview appeared to attenuate overestimation of IFA receipt by >90 tablets, though this estimate included the null (ARR: 0.56 (95% CI 0.31, 1.01). The presence of a husband was less common (7%) and was significantly associated with the overestimation of ANC visits by one, two and three visits. The presence of a friend was significantly associated with overestimation of the receipt of multiple types of ANC services including information about calcium, managing nausea, not drinking alcohol and not smoking or using tobacco.

Table 4**.** Presence of others during the interview as a predictor of overestimation of recalled services receipt

	N	Mother in law present ARR[2] (95% CI)	Husband present ARR[2] (95% CI)	Friend present ARR[2] (95% CI)	Any adult present ARR[2] (95% CI)
Deworming	401	1.90 (1.18, 3.05)	0.50 (0.13, 1.94)	0.85 (0.41, 1.76)	1.05 (0.66, 1.67)
Receipt or prescribe Ca	396	1.02 (0.45, 2.32)	1.63 (0.52, 5.06)	2.69 (1.30, 5.55)	1.91 (0.92, 3.97)
Counselling on					
Managing nausea	397	1.17 (0.89, 1.54)	1.09 (0.68, 1.76)	1.53 (1.15, 2.04)	1.70 (1.30, 2.22)
Not drinking alcohol	401	0.80 (0.58, 1.11)	1.07 (0.66, 1.74)	1.41 (1.05, 1.90)	1.09 (0.85, 1.39)
Not smoking/ using paan	401	0.71 (0.48, 1.06)	1.22 (0.73, 2.06)	1.55 (1.12, 2.15)	0.98 (0.74, 1.31)
Overestimate IFA receipt					
By >30 tablets	402	0.94 (0.76, 1.17)	0.88 (0.60, 1.29)	0.93 (0.69, 1.25)	0.96 (0.80, 1.15)
By >60 tablets	402	0.86 (0.62, 1.19)	1.13 (0.72, 1.77)	0.89 (0.58, 1.37)	0.95 (0.73, 1.24)
By >90 tablets	402	0.56 (0.31, 1.01)	0.90 (0.40, 2.02)	0.96 (0.51, 1.81)	0.73 (0.49, 1.11)
Overestimate # of ANC visits					

For peer review only - http://bmjopen.bmj.com/site/about/guidelines.xhtml

By >1 visit	400	1.28 (0.97, 1.70)	1.48 (1.03, 2.13)	1.18 (0.82, 1.70)	1.33 (1.01, 1.73)
By >2 visits	400	1.32 (0.85, 2.05)	1.97 (1.13, 3.44)	1.24 (0.70, 2.20)	1.57 (1.03, 2.40)
By >3 visits	400	2.07 (1.11, 3.84)	4.19 (2.17, 8.10)	0.43 (0.10, 1.72)	2.26 (1.15, 4.46)

[1]Adjusted risk ratio > 1 indicates greater risk of overestimation of reported services receipt

[3]Adjusted for woman's age (continuous), any education, first pregnancy, and socio-economic status quartile

Women interviewed in the presence of MILs were less accurate in estimating the number of ANC visits but more accurate in recalling the receipt of counselling related to avoiding alcohol consumption in pregnancy. Women tended to be less accurate when estimating the number of ANC visits or receipt of IFA in front of their husbands. (**Table 5**).

Table 5 Presence of others during the interview as a predictor of accuracy of recalled services receipt

	N	Mother in law present RR (95% CI)	Husband present RR (95% CI)	Friend present RR (95% CI)	Any adult present RR (95% CI)
Deworming	401	0.91 (0.79, 1.04)	1.13 (0.99, 1.29)	0.84 (0.57, 1.25)	1.03 (0.93, 1.14)
Receipt or prescribe Ca	396	1.03 (0.94, 1.12)	0.97 (0.81, 1.16)	0.87 (0.74, 1.04)	0.99 (0.92, 1.08)
Counselling on					
Managing nausea	397	0.94 (0.74, 1.19)	0.78 (0.49, 1.25)	0.71 (0.50, 1.02)	0.72 (0.59, 0.87)
Not drinking alcohol	401	1.20 (1.00, 1.45)	1.07 (0.79, 1.45)	0.78 (0.55, 1.10)	1.01 (0.85, 1.20)
Not smoking/ using paan	401	1.14 (0.95, 1.35)	0.91 (0.66, 1.27)	0.76 (0.55, 1.05)	1.04 (0.89, 1.22)
Accuracy of IFA estimate					
Within 30 tablets	402	0.96 (0.73, 1.28)	1.06 (0.68, 1.68)	1.17 (0.84, 1.63)	1.01 (0.80, 1.29)
Within 60 tablets	402	0.91 (0.75, 1.11)	0.76 (0.50, 1.14)	0.96 (0.76, 1.20)	0.94 (0.81, 1.09)
Within 90 tablets	402	1.00 (0.89, 1.12)	0.69 (0.50, 0.96)	0.96 (0.82, 1.13)	0.99 (0.90, 1.10)
Estimate # of ANC visits					
Exactly	400	0.67 (0.41, 1.10)	0.47 (0.16, 1.40)	1.25 (0.75, 2.08)	0.94 (0.65, 1.36)
Within 1 visit	400	0.86 (0.69, 1.08)	0.87 (0.59, 1.27)	1.07 (0.83, 1.37)	1.04 (0.87, 1.24)
Within 2 visits	400	0.85 (0.74, 0.99)	0.76 (0.56, 1.04)	1.07 (0.92, 1.24)	0.93 (0.82, 1.04)

[1]Adjusted risk ratio > 1 indicates greater accuracy of reported services receipt

[3]Adjusted for woman's age (continuous), any education, first pregnancy, and socio-economic status quartile

We also examined how the receipt of help from observers to answer questions during the interview may have affected the accuracy of recall of services **(Supplementary Table 5)**. Assistance from MIL was

For peer review only - http://bmjopen.bmj.com/site/about/guidelines.xhtml

associated with more accurate recall of counselling to not drink alcohol [ARR 1.24 (95% C.I. 1.00, 1.55) but no other services. In contrast, assistance from husbands did not significantly affect the accuracy of recall of any services.

Discussion

Previous studies exploring the validity of self-reported questions about the receipt of ANC care have raised social desirability bias as a possible type of error that might affect coverage estimates.[23,24] To our knowledge, however, this is the first study in the peer-reviewed literature to directly explore whether social desirability bias or the presence of others influences the validity of women's recalled receipt of ANC services. We found that SDS did not significantly predict the accuracy of women's recall of most types of ANC services, except for counselling on the management of nausea. However, the presence of another adult observer during the interview did influence the accuracy of recall. Women that were interviewed with their MIL or husbands present tended to overestimate the number of ANC visits they had received and those interviewed with friends present tended to overestimate the receipt of different types of counselling.

The effects of social desirability bias are complex[25] and socially desirable responses may be driven by either unconscious distortions related to self-deception or impression management related to social norms.[13,26] The potential for social desirability bias to affect questions about nutrition and receipt of ANC services in low- and middle-income countries has only been indirectly explored in the literature. For example, a study conducted in China found that recalled receipt of Hepatitis B blood tests (HBsAb) was much higher than HIV tests despite similar observed coverage and suggested that the difference might be explained by socially desirable responses.[23] Many of the questions that we tested in our study related to whether or not women had received specific nutrition interventions as part of

For peer review only - http://bmjopen.bmj.com/site/about/guidelines.xhtml

ANC visits. A likely explanation for null associations with the SDS is that such questions do not reflect practices that might be perceived negatively (or positively) from the standpoint of social norms.[25] [26]

However, it is unclear why higher scores on the SDS were positively associated with more accurate recall of counselling on management of nausea. One possible explanation is that the counselling focused only on women that complained of nausea and not all women.[5] Thus, it is possible that the socially desirable response to this question was to accurately respond to whether or not counselling had been received, and that these responses were particularly easy to recall because they were related to recollections of nausea.

In contrast to questions about the simple receipt of services during ANC visits, questions about the number of ANC visits or IFA consumption could be more subject to social desirability bias because they relate to a woman's initiative in seeking services or taking supplements and because there is likely positive sentiment around the benefits of services for women and their babies. This could explain the finding that the presence of MILs and/or husbands during the interview was associated with a significantly greater overestimation of the number of ANC visits. Cognitive testing[27] conducted in this same population revealed that women were confused about whether visits to providers outside of health posts including for sonograms should be counted towards their estimate of ANC visits or not, and it is possible that women might be more likely to count such visits when in the presence of other household members.

IFA coverage is an important indicator for global monitoring of nutrition interventions; it is included in the Global Nutrition Monitoring Framework and an indicator for the Countdown to 2030. [28,29] There are global concerns about accuracy of recall of the number of tablets received or consumed during a specific pregnancy, particularly in the DHS or other surveys where respondents may be 2-5 years postpartum. Previous work in Nepal showed that at six months post-partum women's self-

For peer review only - http://bmjopen.bmj.com/site/about/guidelines.xhtml

reported receipt of IFA tended to overestimate the number of supplements that they actually received and that this overestimation was not associated with maternal characteristics including age, education, parity, or SES.[4]

Our study is the first that we are aware of to test a social desirability scale in the context of rural Nepal, though SD scales have been used in urban India[30] and rural Bangladesh.[17] We pretested scale items and made some modifications. The reliability coefficient of our tested scale of 0.58 neared the threshold of 0.60 used to define acceptability, and marginally surpassed that threshold when two items were removed. These values were slightly less than reliability coefficients documented in a study using a lengthier version of the Marlowe-Crowne SDS in four African countries, which varied from 0.63 (Kenya) to 0.80 (Ethiopia).[7] Many of the scale items in our study were positively correlated, however, it is uncertain how well the questions were understood by respondents or the extent to which the scale reflects social desirability in the context of rural Nepal. Additional work to develop and test questions that draw more strongly on local situations and norms would likely further strengthen the ability of the scale to capture social desirability in rural contexts, similar to what has been developed in the Philippines,[29] and could enhance the utility of such scales for use in surveys.

A strength of our study is that we used direct observation to assess the receipt of services in clinics. However, the tool used to assess social desirability has not been validated in a rural context, and was only slightly adapted for comprehension by respondents in our area. One adaptation we made to improve comprehension by the study population was to reformat the SDS tool from statements into questions. Ultimately, however, it is not clear how well the scale was able to capture socially desirable response tendencies in this context. Our study used a recall period of approximately 6 months, which may have facilitated more accurate recall of receipt than typically is assessed in surveys such as the DHS, which currently administers ANC modules to women up to 2-years postpartum. The implications of different recall periods for social desirability bias are unclear. Additionally, the enumerators noted the

For peer review only

For peer review only - http://bmjopen.bmj.com/site/about/guidelines.xhtml

presence of others at any point in the interview, and not specifically during the questions about receipt of services; without more detailed information about observer presence when the questions were asked it is difficult to attribute findings specifically to social desirability bias.

These findings reinforce the guidance often given during survey training to conduct surveys without others present, though doing so in practice is often challenging in rural village settings where norms around privacy differ. As we found that the social desirability scale was not well correlated with overestimation or lack of accurate recall, there was no need to correct for social desirability bias. Given that the generalizability of our study to other contexts including other parts of Nepal is uncertain, we recommend further studies be conducted in other contexts to develop and validate social desirability scales and use them to better understand the effects of social desirability bias on estimates of services use.

Acknowledgements

. The authors thank the study team at the Nepal Nutrition Intervention Project-Sarlahi, who conducted household data collection. The Nepal Nutrition Intervention Project Sarlahi operates with the permission of and under the auspices of the Social Welfare Council in Nepal and in close conjunction with Nepal Netra Jyoti Singh. This work was funded by the Bill & Melinda Gates Foundation INV007332 and OPP1172551

Contributorship statement

Conceived study design: MKM, JK, ATL, RAH; Implemented the study: TPL, SL, SKK, EB, PM, JK; Analyzed the data: ATL; Wrote the paper: ATL; Read, provided edits and approved final manuscript: all authors.

Competing interests

The authors declare no competing interests in relation to this work

For peer review only

Funding

This work was funded by the Bill & Melinda Gates Foundation INV007332 and OPP1172551.

Data sharing statement

Date will be made available on reasonable request

Ethics approval statement

This study was approved by the institutional review board at Johns Hopkins Bloomberg School of Public Health (#8808) and by the Nepal Health Research Council (#441 2018). Participants gave informed consent to participate in the study.

References

1 World Health Organization. *WHO recommendations on antenatal care for a positive pregnancy experience*. World Health Organization 2016.

2 Munos MK, Blanc AK, Carter ED, *et al.* Validation studies for population-based intervention coverage indicators: design, analysis, and interpretation. *J Glob Health* 2018;**8**.

3 Hill Z, Okyere E, Wickenden M, *et al.* What can we learn about postnatal care in Ghana if we ask the right questions? A qualitative study. *Glob Health Action* 2015;**8**:28515.

4 Bryce E, Katz J, Munos MK, *et al.* Validation of Maternal Recall of Iron Folic Supplementation During Antenatal Care in Rural Southern Nepal. *Curr Dev Nutr* 2021;**5**:722–722.

5 Bryce E, Katz J, Heidkamp R, *et al.* Validation of maternal report of nutrition-related interventions and counselling during antenatal care in southern Nepal. *Matern Child Nutr* 2021;:e13303.

6 Stuart GS, Grimes DA. Social desirability bias in family planning studies: a neglected problem. *Contraception* 2009;**80**:108–12.

7 Vu A, Tran N, Pham K, *et al.* Reliability of the Marlowe-Crowne social desirability scale in Ethiopia, Kenya, Mozambique, and Uganda. *BMC Med Res Methodol* 2011;**11**:1–7.

8 Adida CL, Ferree KE, Posner DN, *et al.* Who’s asking? Interviewer coethnicity effects in African survey data. *Comp Polit Stud* 2016;**49**:1630–60.

For peer review only

For peer review only - http://bmjopen.bmj.com/site/about/guidelines.xhtml

9 Crowne DP, Marlowe D. A new scale of social desirability independent of psychopathology. *J Consult Psychol* 1960;**24**:349.

10 Cagasan Jr L. The development and validation of a Filipino social desirability scale. *Philipp J Psychol* 2016;**49**:19–42.

11 Reynolds WM. Development of reliable and valid short forms of the Marlowe-Crowne Social Desirability Scale. *J Clin Psychol* 1982;**38**:119–25.

12 Paulhus DL. Measurement and control of response bias. 1991.

13 Larson RB. Controlling social desirability bias. *Int J Mark Res* 2019;**61**:534–47.

14 Näher A-F, Krumpal I. Asking sensitive questions: the impact of forgiving wording and question context on social desirability bias. *Qual Quant* 2012;**46**:1601–16.

15 Westerterp KR, Goris AH. Validity of the assessment of dietary intake: problems of misreporting. *Curr Opin Clin Nutr Metab Care* 2002;**5**:489–93.

16 Nguyen PH, Kim SS, Sanghvi T, *et al.* Integrating nutrition interventions into an existing maternal, neonatal, and child health program increased maternal dietary diversity, micronutrient intake, and exclusive breastfeeding practices in Bangladesh: results of a cluster-randomized program evaluation. *J Nutr* 2017;**147**:2326–37.

17 Menon P, Nguyen PH, Saha KK, *et al.* Impacts on breastfeeding practices of at-scale strategies that combine intensive interpersonal counselling, mass media, and community mobilization: results of cluster-randomized program evaluations in Bangladesh and Viet Nam. *PLoS Med* 2016;**13**:e1002159.

18 Ministry of Health, Nepal; New ERA; and ICF. Nepal Demographic and Health Survey 2016. Kathmandu, Nepal: : Ministry of Health, Nepal 2017.

19 Adhikari M, Chalise B, Bista B, *et al.* Sociodemographic correlates of antenatal care visits in Nepal: results from Nepal Demographic and Health Survey 2016. *BMC Pregnancy Childbirth* 2020;**20**:1–10.

20 Mullany LC, Katz J, Li YM, *et al.* Breast-feeding patterns, time to initiation, and mortality risk among newborns in southern Nepal. *J Nutr* 2008;**138**:599–603.

21 World Health Organization. WHO antenatal care randomized trial: manual for the implementation of the new model. World Health Organization 2002.

22 Spiegelman D and Hertzmark E. Easy SAS calculations for risk or prevalence ratios and differences. American Journal of Epidemiology 2005;**162.3**: 199-200.

23 Liu L, Li M, Yang L, *et al.* Measuring coverage in MNCH: a validation study linking population survey derived coverage to maternal, newborn, and child health care records in rural China. *PLoS One* 2013;**8**:e60762.

24 Benova L, Tunçalp Ö, Moran AC, *et al.* Not just a number: examining coverage and content of antenatal care in low-income and middle-income countries. *BMJ Glob Health* 2018;**3**:e000779.

For peer review only

For peer review only - http://bmjopen.bmj.com/site/about/guidelines.xhtml

25 Fisher RJ, Katz JE. Social-desirability bias and the validity of self-reported values. *Psychol Mark* 2000;**17**:105–20.

26 Crowne DP, Crowne DP, Marlowe D. *The approval motive: Studies in evaluative dependence*. New York: Wiley 1964.

27 Thorne-Lyman AL, Lama TP, Heidkamp RA, *et al.* Cognitive testing of questions about antenatal care and nutrition interventions in southern Nepal. *Soc Sci Med* In Press.

28 World Health Organization. Developing and validating an iron and folic acid supplementation indicator for tracking progress towards global nutrition monitoring framework targets: final report June 2018. 2018.

29 Boerma T, Requejo J, Victora CG, *et al.* Countdown to 2030: tracking progress towards universal coverage for reproductive, maternal, newborn, and child health. *The Lancet* 2018;**391**:1538–48.

30 Ranjan A, George TS. Evaluating social desirability bias in assessment. *Indian J Health Wellbeing* 2014;**5**:948.

For peer review only

For peer review only - http://bmjopen.bmj.com/site/about/guidelines.xhtml

Supplementary Materials

Supplementary Figure 1. Study flow chart

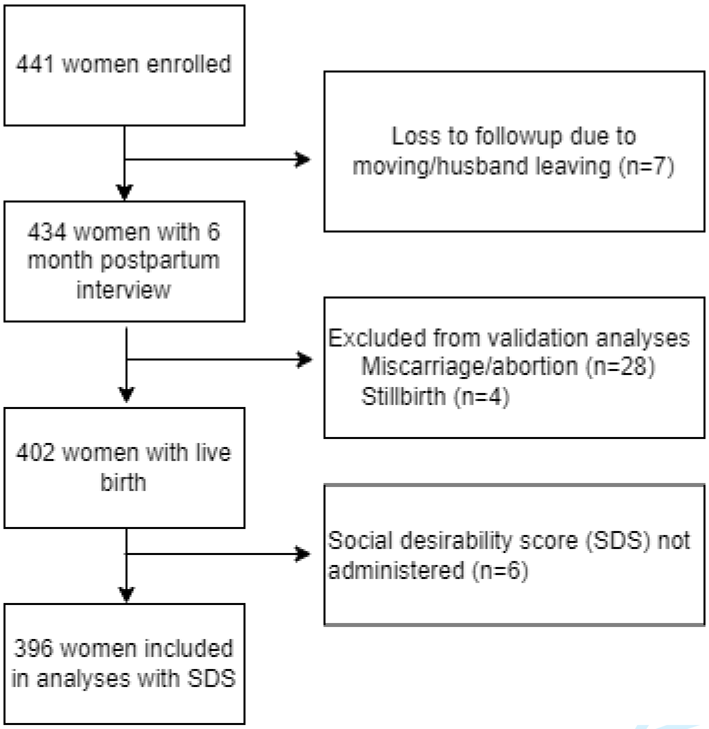

view only

Supplementary Tables

Supplementary Table 1. Questions included in the survey and their origins[1]

Question	Responses	Origin/notes
21. How many times did you receive antenatal care during this pregnancy?	Number of times:<br>01-15 times (go to 21.a.)<br>99= Don't know (go to 22)	2016 Demographic and Health Survey, DHS-8
23. During this pregnancy, were you given or did you buy any iron/folic acid tablets?<br><br>Show photo of tablets	0= No (go to 24)<br>1= Yes (go to 23.a.)<br>9= Don't Know (go to 24)	2016 Demographic and Health Survey, DHS-8
23.a. During the whole pregnancy, for how many days did you take the tablets?<br>If answer is not numeric, probe for approximate number of days	Days 000-270<br>999=Don't know	2016 Demographic and Health Survey, DHS-8
27. During this pregnancy, at any antenatal visit, did you receive any information about nutrition or diet?	0= No (go to 28)<br>1= Yes (go to 27.a.)<br>9= Don't Know (go to 28)	Original question
27.b. What information or messages did you receive during your pregnancy about nutrition or diet?<br><br>First allow respondent to provide any answers and check all that apply<br><br>Then probe to identify each response and record all mentioned<br>[ask each answer individually]	Were you told to…<br><br>□ Eat more (quantity)<br>□ Eat a variety of foods rich with iron, vitamin A or C<br>□ Take iron tablets (IFA's)<br>□ Take calcium tablets<br>□ How to manage nausea/vomiting<br>□ Other (specify) ______<br><br>0= No<br>1= Yes, without prompting<br>2= Yes, with prompting<br>9= Don't know	Original question

[1]These questions were administered to respondents approximately 6 months after delivery, asking them to recall services received during the most recent pregnancy. The responses column lists the possible response categories for each question and the origin column describes the source of the question.

For peer review only

Supplementary Table 2. List of questions included from the short form Marlowe Crown social desirability index, inclusion in the score

Code	Question	% Yes	Negative key[1]
SM1	Do you sometimes find it hard to go on with your work if you are not encouraged?	39.6	No
SM2	Do you sometimes feel resentful when you don't get your way?	10.0	No
SM3	Do you occasionally give up doing something because you don't think you have the ability	31.1	No
SM4	Are there any times when you felt like rebelling against people in authority even though you knew they were right?	59.9	No
SM5	Are you always a good listener no matter who you are talking to?	21.9	Yes
SM6	Are there any occasions when you took advantage of someone?	89.1	No
SM7	When you make a mistake are you always willing to admit it?	64.7	Yes
SM8	Are you always courteous, even to people who are disagreeable/not pleasant?	19.5	Yes
SM9	Have you ever been irked when people expressed ideas very different from your own?	14.4	No
SM10	Are there any times when you were quite jealous of the good fortune of others?	85.1	No
SM11	Do you sometimes get irritated/annoyed by people who ask you to do something for them?	27.6	No
SM12	Have you ever deliberately said something that hurt someone's feelings	89.5	No

For peer review only

Supplementary Table 3. Correlations between questions used for assessment of the social desirability

		SM1	SM2	SM3	SM4	SM5	SM6	SM7	SM8	SM9	SM10	SM11	SM12
SM1	Do you sometimes find it hard to go on with your work if you are not encouraged?	1.00											
SM2	Do you sometimes feel resentful when you don't get your way?	0.31 ***	1.00										
SM3	Do you occasionally give up doing something because you don't think you have the ability	0.07	0.08	1.00									
SM4	Are there any times when you felt like rebelling against people in authority even though you knew they were right?	-0.03	0.10 *	-0.08	1.00								
SM5	Are you always a good listener no matter who you are talking to?	0.15 **	0.16 **	0.03	0.22 ***	1.00							
SM6	Are there any occasions when you took advantage of someone?	.02	.01	-0.01	0.11 *	-0.03	1.00						
SM7	When you make a mistake are you always willing to admit it?	.08	0.04	-0.12 *	0.36 ***	0.23 ***	-0.04	1.00					
SM8	Are you always courteous, even to people who are disagreeable/not pleasant?	.21 ***	0.21 ***	0.03	0.03	0.38 ***	-0.03	0.21 ***	1.00				
SM9	Have you ever been irked when people expressed ideas very different from your own?	.20 ***	0.34 ***	-0.03	0.15 **	0.16 **	0.07	0.13 *	0.32 ***	1.00			
SM10	Are there any times when you were quite jealous of the good fortune of others?	0.16 **	0.07	0.04	0.15 *	0.07	0.06	0.15 **	0.05	0.11 *	1.00		
SM11	Do you sometimes get irritated/annoyed by people who ask you to do something for them?	.29 ***	0.34 ***	0.01	0.15 **	0.28 ***	-0.02	0.21 ***	0.32 ***	0.29 ***	0.15 **	1.00	
SM12	Have you ever deliberately said something that hurt someone's feelings	-0.11 *	-0.02	0.005	0.02	0.07	0.17 **	-0.04	-0.02	-0.07	0.06	0.03	1.00

***p<0.0001, **p<0.01 *p<0.05

For peer review only

Supplementary Table 4. Characteristics of women and households by presence of others during the interview

	No other adults present (n=195)	Any other adult present (n=207)	Husband present (n=28)	Mother in law present (n=98)	Friend present (n=50)	Overall (n=402)
Women's age, years (mean, sd)	22.7 (4.3)	22.3 (4.1)	20.6 (2.9)	21.8 (3.7)	23.4 (4.8)	22.5 (4.2)
# Observed ANC visits	5.0 (2.6)	4.3 (2.3)	4. 1 (2.1)	4.4 (2.5)	4.4 (2.3)	4.7 (2.5)
Socioeconomic status quartile %						
1 (Bottom)	34.9	38.7	32.1	36.7	40.0	36.8
2	18.0	17.4	21.4	17.4	16.0	17.7
3	31.3	31.4	32.1	29.6	38.0	31.3
4 (Top)	15.9	12.6	14.3	16.3	6.0	14.2
Any prior live births (%)	70.3	67.2	60.7	59.2	74.0	68.7
Any formal education (%)	42.1	38.6	42.9	44.9	24.0	40.3
Trimester at enrollment						
1-3 months	46.2	38.7	28.6	40.8	34.0	42.3
4-6 months	51.8	59.9	71.4	57.1	66.0	56.0
7-9 months	2.1	1.5	0	2.0	0	1.7
Got help during interview*	0	52.0	50.0	48.1	32.7	19.9

For peer review only

Supplementary Table 5. Receipt of help during the interview and accuracy of recall of services

	Received help during interview from another adult ARR (95% CI)	Received help from MIL during the interview ARR (95% CI)
Deworming	0.97 (0.78, 1.20)	0.94 (0.78, 1.13)
Receipt of Ca or Ca info	0.99 (0.82, 1.20)	1.01 (0.90, 1.13)
Counseling on		
Nutrition	0.97 (0.85, 1.11)	No convergence
Eating more	0.93 (0.77, 1.12)	No convergence
Diverse diet	No convergence	No convergence
Managing nausea	1.02 (0.70, 1.49)	1.18 (0.91, 1.53)
Not drinking *paan* alcohol	0.94 (0.64, 1.38)	1.24 (1.00, 1.55)
Not smoking/ using	0.88 (0.60, 1.29)	1.14 (0.92, 1.41)
Accuracy of IFA estimate		
Within 30 tablets	1.08 (0.69, 1.69)	1.16 (0.81, 1.64)
Within 60 tablets	1.04 (0.80, 1.36)	1.07 (0.85, 1.34)
Within 90 tablets	1.05 (0.89, 1.24)	1.09 (0.96, 1.25)
Estimate number of ANC visits		
Exactly	0.47 (0.16, 1.38)	0.76 (0.39, 1.49)
Within 1 visit	0.86 (0.57, 1.29)	0.81 (0.58, 1.13)
Within 2 visits	0.98 (0.78, 1.23)	1.02 (0.86, 1.21)

For peer review only

STROBE Statement—checklist of items that should be included in reports of observational studies

	Item No	Recommendation	Page No
**Title and abstract**	1	(*a*) Indicate the study's design with a commonly used term in the title or the abstract	2
		(*b*) Provide in the abstract an informative and balanced summary of what was done and what was found	2
**Introduction**			
Background/rationale	2	Explain the scientific background and rationale for the investigation being reported	4
Objectives	3	State specific objectives, including any prespecified hypotheses	5
**Methods**			
Study design	4	Present key elements of study design early in the paper	6-7
Setting	5	Describe the setting, locations, and relevant dates, including periods of recruitment, exposure, follow-up, and data collection	6
Participants	6	(*a*) *Cohort study*—Give the eligibility criteria, and the sources and methods of selection of participants. Describe methods of follow-up<br>*Case-control study*—Give the eligibility criteria, and the sources and methods of case ascertainment and control selection. Give the rationale for the choice of cases and controls<br>*Cross-sectional study*—Give the eligibility criteria, and the sources and methods of selection of participants	8
		(*b*) *Cohort study*—For matched studies, give matching criteria and number of exposed and unexposed<br>*Case-control study*—For matched studies, give matching criteria and the number of controls per case	
Variables	7	Clearly define all outcomes, exposures, predictors, potential confounders, and effect modifiers. Give diagnostic criteria, if applicable	6-9
Data sources/ measurement	8*	For each variable of interest, give sources of data and details of methods of assessment (measurement). Describe comparability of assessment methods if there is more than one group	6-8
Bias	9	Describe any efforts to address potential sources of bias	Entire paper
Study size	10	Explain how the study size was arrived at	7
Quantitative variables	11	Explain how quantitative variables were handled in the analyses. If applicable, describe which groupings were chosen and why	9
Statistical methods	12	(*a*) Describe all statistical methods, including those used to control for confounding	9
		(*b*) Describe any methods used to examine subgroups and interactions	na
		(*c*) Explain how missing data were addressed	9
		(*d*) *Cohort study*—If applicable, explain how loss to follow-up was addressed<br>*Case-control study*—If applicable, explain how matching of cases and controls was addressed<br>*Cross-sectional study*—If applicable, describe analytical methods taking account of sampling strategy	9, Supp fig 1

For peer review only - http://bmjopen.bmj.com/site/about/guidelines.xhtml
For peer review only

(*e*) Describe any sensitivity analyses	

Continued on next page

For peer review only

For peer review only - http://bmjopen.bmj.com/site/about/guidelines.xhtml

**Results**			
Participants	13*	(a) Report numbers of individuals at each stage of study—eg numbers potentially eligible, examined for eligibility, confirmed eligible, included in the study, completing follow-up, and analysed	Sup Fig 1, 10
		(b) Give reasons for non-participation at each stage	Supp fig 1
		(c) Consider use of a flow diagram	Supp fig 1
Descriptive data	14*	(a) Give characteristics of study participants (eg demographic, clinical, social) and information on exposures and potential confounders	Table 1; supp table 4
		(b) Indicate number of participants with missing data for each variable of interest	tables
		(c) *Cohort study*—Summarise follow-up time (eg, average and total amount)	Page 8
Outcome data	15*	*Cohort study*—Report numbers of outcome events or summary measures over time	*tables*
		Case-control study—Report numbers in each exposure category, or summary measures of exposure	
		Cross-sectional study—Report numbers of outcome events or summary measures	
Main results	16	(*a*) Give unadjusted estimates and, if applicable, confounder-adjusted estimates and their precision (eg, 95% confidence interval). Make clear which confounders were adjusted for and why they were included	No room for unadjusted.
		(*b*) Report category boundaries when continuous variables were categorized	9
		(*c*) If relevant, consider translating estimates of relative risk into absolute risk for a meaningful time period	na
Other analyses	17	Report other analyses done—eg analyses of subgroups and interactions, and sensitivity analyses	Supp tables
**Discussion**			
Key results	18	Summarise key results with reference to study objectives	10-11
Limitations	19	Discuss limitations of the study, taking into account sources of potential bias or imprecision. Discuss both direction and magnitude of any potential bias	13-14
Interpretation	20	Give a cautious overall interpretation of results considering objectives, limitations, multiplicity of analyses, results from similar studies, and other relevant evidence	13-14,
Generalisability	21	Discuss the generalisability (external validity) of the study results	14
**Other information**			
Funding	22	Give the source of funding and the role of the funders for the present study and, if applicable, for the original study on which the present article is based	1

*Give information separately for cases and controls in case-control studies and, if applicable, for exposed and unexposed groups in cohort and cross-sectional studies.

Note: An Explanation and Elaboration article discusses each checklist item and gives methodological background and published examples of transparent reporting. The STROBE checklist is best used in conjunction with this article (freely available on the Web sites of PLoS Medicine at http://www.plosmedicine.org/, Annals of Internal Medicine at http://www.annals.org/, and Epidemiology at http://www.epidem.com/). Information on the STROBE Initiative is available at www.strobe-statement.org.

For peer review only

For peer review only - http://bmjopen.bmj.com/site/about/guidelines.xhtml
